# A *SOC1-like* gene *MtSOC1a* promotes flowering and primary stem elongation in Medicago

**DOI:** 10.1093/jxb/ery284

**Published:** 2018-07-26

**Authors:** Mauren Jaudal, Lulu Zhang, Chong Che, Guifen Li, Yuhong Tang, Jiangqi Wen, Kirankumar S Mysore, Joanna Putterill

**Affiliations:** 1 Flowering Lab, School of Biological Sciences, University of Auckland, Auckland, New Zealand; 2 Noble Research Institute, LLC, Ardmore, OK, USA

**Keywords:** Cell elongation, flowering, *FTa1*, gibberellins, internode, Medicago, *MtSOC1a*, *MtSOC1b*, *MtSOC1c*, primary shoot axis elongation

## Abstract

Medicago flowering, like that of Arabidopsis, is promoted by vernalization and long days, but alternative mechanisms are predicted because Medicago lacks the key regulators CO and FLC. Three Medicago *SOC1-like* genes, including *MtSOC1a*, were previously implicated in flowering control, but no legume *soc1* mutants with altered flowering were reported. Here, reverse transciption–quantitative PCR (RT–qPCR) indicated that the timing and magnitude of *MtSOC1a* expression was regulated by the flowering promoter *FTa1*, while *in situ* hybridization indicated that *MtSOC1a* expression increased in the shoot apical meristem during the floral transition. A *Mtsoc1a* mutant showed delayed flowering and short primary stems. Overexpression of *MtSOC1a* partially rescued the flowering of *Mtsoc1a*, but caused a dramatic increase in primary stem height, well before the transition to flowering. Internode cell length correlated with stem height, indicating that *MtSOC1a* promotes cell elongation in the primary stem. However, application of gibberellin (GA_3_) caused stem elongation in both the wild type and *Mtsoc1a*, indicating that the mutant was not defective in gibberellin responsiveness. These results indicate that *MtSOC1a* may function as a floral integrator gene and promotes primary stem elongation. Overall, this study suggests that apart from some conservation with the Arabidopsis flowering network, *MtSOC1a* has a novel role in regulating aspects of shoot architecture.

## Teaser

Overexpression of Medicago *SOC1a* leads to precocious primary shoot axis elongation.

## Introduction

The timing of flowering is a critical agronomic trait in plants because of its major effects on reproductive and vegetative productivity (Julier *et al.*, 2007; Jung and Müller, 2009; Yeoh *et al.*, 2011; Tadege *et al.*, 2015). Plants have evolved sophisticated flowering gene networks that integrate different internal and external signals before making the decision to flower (Putterill *et al.*, 2004; Srikanth and Schmid, 2011). In *Arabidopsis thaliana* (Arabidopsis), the MADS box transcription factor gene *SUPPRESSOR OF OVEREXPRESSION OF CONSTANS 1* (*SOC1*) and *FLOWERING LOCUS T* (*FT*) function as key floral integrator genes that promote the transition to flowering (Lee and Lee, 2010; Andrés and Coupland, 2012; Immink *et al.*, 2012; Tao *et al.*, 2012). In inductive long-day (LD) conditions, the FT florigen moves via the phloem to the shoot apex, where it rapidly activates *SOC1* expression (Michaels *et al.*, 2005; Turck *et al.*, 2008; Berry and Dean, 2015; Putterill and Varkonyi-Gasic, 2016). SOC1 interacts with AGAMOUS-LIKE 24 (AGL24) and FRUITFULL (FUL) to activate *LEAFY* (*LFY*), which then triggers the expression of the floral meristem identity gene *APETALA 1* (*AP1*) and thus floral commitment and the ensuing development of flowers on the flanks of the shoot apical meristem (Lee and Lee, 2010; Balanzà *et al.*, 2014).

The Arabidopsis *soc1* mutant is late flowering, and *SOC1* overexpression causes Arabidopsis to flower early (Borner *et al.*, 2000; Lee *et al.*, 2000; Onouchi *et al.*, 2000; Samach *et al.*, 2000). In addition, analysis of *soc1 ful* double mutants uncovered an overlapping role of these genes in acceleration of flowering time in LDs, floral commitment, meristem determinacy, and in suppressing perennial characters in Arabidopsis (Melzer *et al.*, 2008). *AtSOC1* plays a redundant role with *AGL24* and *SHORT VEGETATIVE PHASE* (*SVP*) in promoting the proliferation of the floral primordium during the early stages of flower development. It is then repressed, but transcript is detected at low levels later in flower development (Immink *et al.*, 2012). Genome-wide studies indicate that AtSOC1 binds to and regulates a large number of flowering regulators including floral homeotic genes and *AP2-like* floral repressors. SOC1 also directly represses its own expression and that of its negative regulators such as *SVP* (Immink *et al.*, 2012; Tao *et al.*, 2012; Mateos *et al.*, 2015).

SOC1 is in the TM3 clade of MIKC^c^ MADS proteins which was present in all 27 flowering plant genomes analysed (Gramzow and Theißen, 2015), indicative of its general importance. In rice, the *SOC1-like* gene *OsMADS50* promotes flowering while *OsMADS56* represses flowering when overexpressed (Lee *et al.*, 2004; Komiya *et al.*, 2009; Ryu *et al.*, 2009). The *FvSOC1* gene also represses flowering in woodland strawberry (Mouhu *et al.*, 2013). In maize, *ZmMADS1* promotes the transition to flowering (Alter *et al.*, 2016) and, in petunia, silencing of its three *SOC1-like* genes delays flowering (Preston *et al.*, 2014). Several *SOC1-like* genes promote flowering when overexpressed, including orchid *DoSOC1* that accelerates flowering in Arabidopsis and orchid (Ding *et al.*, 2013), cotton *GhSOC1* (Zhang *et al.*, 2016) that promotes flowering in Arabidopsis, and tree peony *PsSOC1* that accelerates tobacco flowering (Wang *et al.*, 2015).

In legumes, a *SOC1-like* gene, *GmGAL1/GmSOC1*, from the tropical short-day (SD) plant soybean accelerates flowering when overexpressed in Arabidopsis (Zhong *et al.*, 2012) and *Lotus corniculatus* (Na *et al.*, 2013). However, loss-of-function *soc1* mutations have not yet been associated with altered flowering time for any legume (Fudge *et al.*, 2018). We are studying flowering time regulation in the temperate-climate reference legume *Medicago truncatula* (Medicago), using its genomic resources including a complete genome sequence and *Tnt1* retroelement insertion mutant populations (Tadege *et al.*, 2009; Young *et al.*, 2011; Putterill *et al.*, 2013). Although, like winter annual Arabidopsis, Medicago flowering is induced by vernalization followed by LD photoperiods (Clarkson and Russell, 1975), novel mechanisms of flowering time control are probably present because unlike Arabidopsis, Medicago lacks both the FLOWERING LOCUS C (FLC) clade of floral repressors and the CONSTANS (CO)-mediated photoperiodic switch function (Putterill *et al.*, 2013; Weller and Ortega, 2015).

To dissect the Medicago flowering network, we isolated Medicago flowering time mutants including the early flowering *spring* mutants (Jaudal *et al.*, 2013; Putterill *et al.*, 2013; Yeoh *et al.*, 2013) and identified flowering time regulators by reverse genetic approaches (Laurie *et al.*, 2011; Jaudal *et al.*, 2014, 2015, 2016). The Medicago *FT-like* gene, *FTa1*, strongly promotes the transition to flowering in Medicago and is a major integrator of the floral inductive signals of vernalization and LDs (Laurie *et al.*, 2011). We also uncovered an important role for the predicted Polycomb complex member MtVERNALISATION2 (MtVRN2) as a flowering repressor, repressing *FTa1* before vernalization (Jaudal *et al.*, 2016). Thus, MtVRN2 has a different function from Arabidopsis VRN2 which stably represses *FLC* after vernalization (Berry and Dean, 2015).

Our previous results and other studies (Fudge *et al.*, 2018) also implicated a *SOC1-like* gene, *MtSOC1a*, as a target of *FTa1* in flowering in Medicago. Transcripts of *MtSOC1a* accumulated to higher levels in early flowering transgenic Medicago plants overexpressing *FTa1* and in the Medicago *spring* and *Mtvrn2* mutants which precociously expressed *FTa1* (Jaudal *et al.*, 2013, 2015, 2016; Yeoh *et al.*, 2013). Interestingly, soybean *GmSOC1* gene transcript levels were also elevated in early flowering soybean overexpressing an *FT-like* gene, *GmFT2a* (Nan *et al.*, 2014).

Here, we focused on characterizing and functionally analysing the role of *MtSOC1a* by profiling its temporal and spatial expression, examining Arabidopsis and Medicago transgenic plants ectopically expressing *MtSOC1a* and determining the phenotype of a *Mtsoc1a Tnt1* mutant. These analyses indicated that apart from its capacity to promote flowering, *MtSOC1a* also promotes elongation of the primary shoot axis of Medicago, a function that has not been shown with *SOC1-like* genes before. We also profiled the expression of the other two Medicago *MtSOC1* genes, *MtSOC1b* and *MtSOC1c*, and found that they were strongly dependent on *MtFTa1* for their expression as previously observed (Fudge *et al.*, 2018), but had little effect on wild-type Arabidopsis flowering time when overexpressed.

## Materials and methods

### Bioinformatics

Reciprocal BLASTP searches were used to identify Medicago TM3 clade MADS box proteins in the JCVI Medicago genome (Mt4.0 http://blast.jcvi.org/Medicago-Blast/index.cgi). Additional SOC1 protein sequences were obtained from Hecht *et al.* (2005) and Gramzow and Theißen (2015) and from JCVI, TAIR (http://www.arabidopsis.org/), NCBI (http://www.ncbi.nlm.nih.gov/), and PHYTOZOME v10 (http://phytozome.jgi.doe.gov) (Goodstein *et al.*, 2012). *In silico* analysis of normalized RNA sequencing (RNA Seq) data was from the MedicMine *Medicago truncatula* genome database (http://medicmine.jcvi.org/medicmine/begin.do). Medicago *Tnt1* lines were identified by screening the FST database (https://medicago-mutant.noble.org/mutant/blast/blast.php).

### Plant growth conditions and materials

Growth in controlled environment conditions under cool white fluorescent lights at ~22 ^o^C in LDs (16 h of light/8 h of dark) or SDs (8 h light/16 h dark) with or without prior vernalization was done as described previously (Laurie *et al.*, 2011; Yeoh *et al.*, 2013; Jaudal *et al.*, 2015).

*Medicago truncatula* (Medicago) wild-type R108_C3 (R108) (Trinh *et al.*, 1998), the *fta1 Tnt1* insertion mutant NF3307, and the *FTa1* overexpression line *35S:FTa1* in R108 were previously reported (Laurie *et al.*, 2011). *Tnt1* insertion mutants (this work) for *MtSOC1a* (NF1705) and *MtSOC1b* (NF7041) in the R108 background were obtained from the Noble Research Institute (Ardmore, OK, USA). The *Mtsoc1a* (NF1705) homozygous mutant was backcrossed once to wild-type R108 and the segregating F_2_ population were used for linkage analysis and F_3_ homozygous mutants in some experiments. The *35S:MtSOC1a* transgenic plants in the *Mtsoc1a* mutant background (NF1705) and *Mtsoc1a* mutant (NF1705) regeneration controls (RCs) were generated in this work. The *35S:MtSOC1a* (*5-1*) transgenic line in the *Mtsoc1a* mutant (NF1705) background was also crossed with wild-type R108, and the F_1_ and segregating populations were analysed. Genotyping was done using gene-specific and *Tnt1* primers (see Supplementary Table S1 at *JXB* online).

Agrobacterium EHA105 with the *35S:MtSOC1a* construct (see below) was used to transform leaf tissues of *Mtsoc1a.* T_0_ transformants were selected using phosphinothricin and plants were regenerated via somatic embryogenesis as described previously (Cosson *et al.*, 2006; Laurie *et al.*, 2011). Transformed plants were rescued to soil and escapes were eliminated by spraying with Basta herbicide, and genotyping. Flowering time was measured in days after planting and the number of nodes on the primary axis at the time the plant flowered. The length of the primary shoot axis (main stem) was measured from the monofoliate leaf node to the uppermost shoot apical bud.

Epidermal leaf peels were obtained by lightly nicking the stem using a double-edged razor blade, then peeling using fine forceps. Peels were mounted in water on slides and photographed using bright field microscopy (Leica DMR). Plants were sprayed with 10 μM or 100 μM gibberellin (dissolved in 0.05% ethanol and 0.02% Tween) or control spray (0.05% ethanol and 0.02% Tween) twice per week unless otherwise specified.

*Arabidopsis thaliana* (Arabidopsis) wild-type Columbia (Col) was used. Transgenic plants overexpressing *MtSOC1* genes (*35S:MtSOC1a–c*) or the Medicago *AP1-like* gene *MtPIM* (Medtr8g066260, *35S:MtPIM*) were generated by inserting the R108 cDNAs into pB2GW7 (Karimi *et al.*, 2002). Agrobacterium GV3101 containing the pB2GW7 vectors was applied using floral dipping, and transgenic T_1_ and T_2_ plants were selected by Basta spraying and genotyping as described previously (Martinez-Trujillo *et al.*, 2004; Jaudal *et al.*, 2015). Flowering time of Arabidopsis plants was measured as the total number of rosette and cauline leaves at flowering.

### RNA extraction and reverse transcriptase–quantitative PCR (RT–qPCR) analysis

RT–qPCR was performed on Arabidopsis and Medicago samples using an oligo(dT) primer and SYBR Green PCR Master Mix on an Applied Biosystems 7900HT Sequence Detection System (Laurie *et al.*, 2011; Yeoh *et al.*, 2013; Jaudal *et al.*, 2015). Each data point is derived from the mean of three biological replicates harvested in parallel, with each replicate consisting of a pool of tissues from at least three independent plants. Leaf and shoot apical samples (hand dissected by eye to remove leaves) were harvested separately. The identity of the PCR amplicons was checked by DNA sequencing. Gene expression was normalized to *PROTEIN PHOSPHATASE 2A* (*PP2A*) (Medtr6g084690) and *At2g32170* for Arabidopsis. To calculate relative gene expression, the 2^-ΔΔ**CT**^ method was used (Livak and Schmittgen, 2001) with modifications (Bookout and Mangelsdorf, 2003). The sample with the highest gene expression relative to *PP2A* is given a value of 1 and the gene expression of the other samples is calculated relative to that. Primers are shown in Supplementary Table S1.

### *In situ* hybridization

R108 seeds were vernalized at 4 °C for 7 d and then grown in LDs with day and night temperatures of 22 °C and 18 °C, respectively. The plants flowered between 4 and 6 weeks. Shoot apices at both vegetative and reproductive stages of wild-type R108 plants (4 weeks old) were fixed and *in situ* hybridization was performed as described previously (Zhou *et al.*, 2010). A 285 bp cDNA fragment from the non-conserved region of *MtSOC1a* was used as a probe for hybridization.

## Results

### *MtSOC1a* encodes one of four TM3 clade MADS proteins in Medicago

**Fig. 1.**
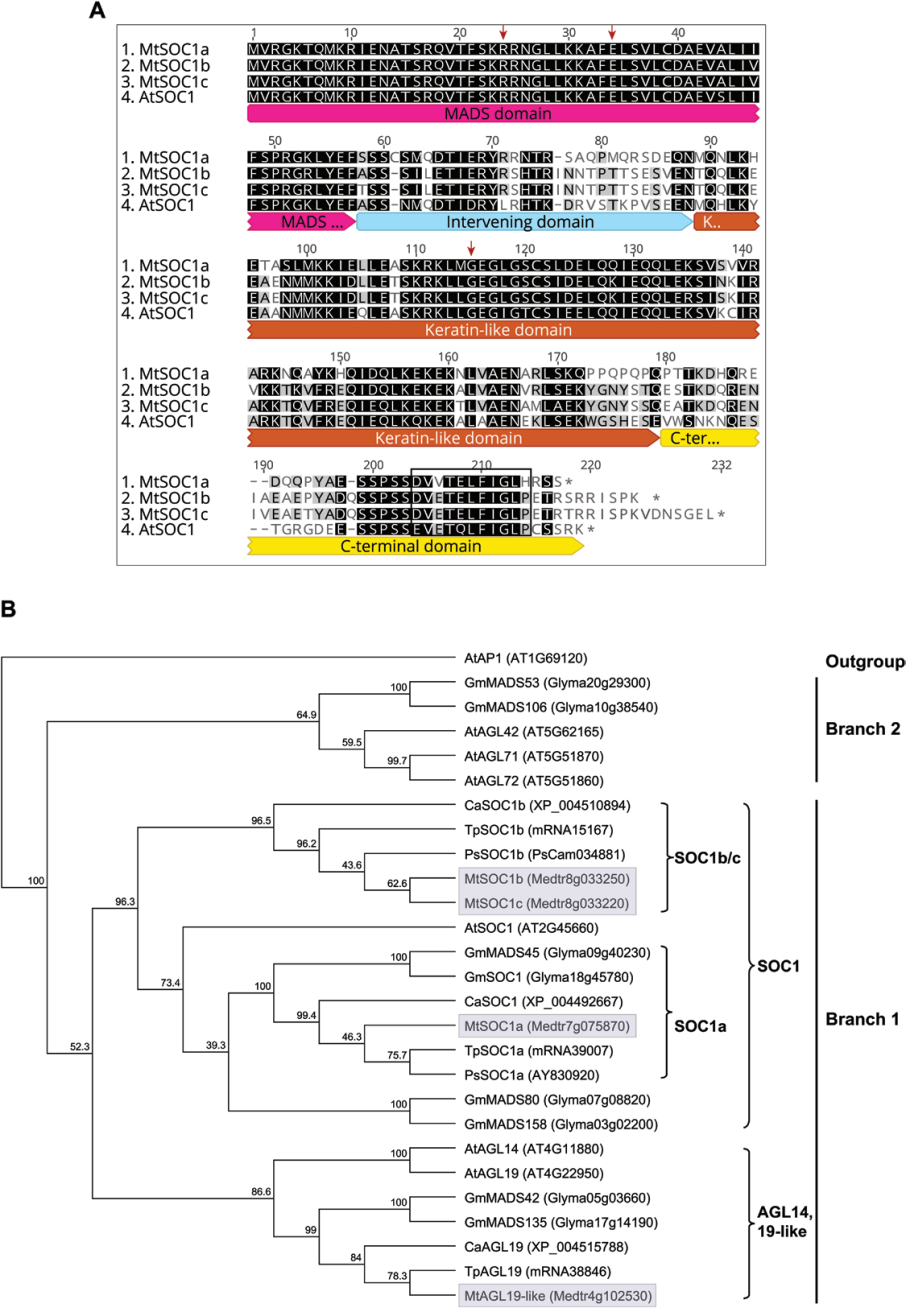
Alignment and phylogenetic analysis of MtSOC1-like proteins. (A) Protein sequence alignment of AtSOC1, MtSOC1a, MtSOC1b, and MtSOC1c indicating the conserved MADS (M), intervening (I), and Keratin-like (K) domains of the MIKC MADS box proteins. The SOC1 motif (EVETQLFIGLP) (Ding *et al.*, 2013) is boxed and the three functional residues identified by previous functional analysis of AtSOC1 (Wang *et al.*, 2015) are marked with arrows. Identical and similar residues are highlighted in black. (B) A consensus phylogenetic tree based on the MIK domains of TM3 members from Arabidopsis, soybean, and the temperate legumes, Medicago, pea, chick pea, and red clover rooted on Arabidopsis APETALA1 (AtAP1). The tree was generated using the Neighbor–Joining (NJ) method via bootstrap resampling with a support threshold of 39%. The numbers indicate the bootstrap values based on 1000 replicates. The branch numbering is from Dorca-Fornell *et al.* (2011). The four Medicago proteins are highlighted in grey. At, *Arabidopsis thaliana*; Ca, *Cicer arietinum* (chick pea); Gm, *Glycine max* (soybean); Mt, *Medicago truncatula*; Ps, *Pisum sativum* (pea); Tp, *Trifolium pratense* (red clover).

A previous phylogenetic analysis of TM3 MIKC^c^ MADS proteins, that did not include Medicago proteins, indicated that an ancient gene duplication gave rise to two main branches: branch 1 included Arabidopsis SOC1, AGL14, and AGL19; and branch 2 had AGL42, 71, and 72, all of which are implicated in flowering regulation and other processes (Dorca-Fornell *et al.*, 2011). Our analysis of the Mt4.0 genome (Tang *et al.*, 2014) identified four members; the three SOC1-like proteins MtSOC1a, (Medtr7g075870), MtSOC1b (Medtr8g033250), and MtSOC1c (Medtr8g033220) (Hecht *et al.*, 2005; Gramzow and Theißen, 2015; Fudge *et al.*, 2018), and Medtr4g102530, more similar to AGL19, but not its top hit in reciprocal BLAST. MtSOC1a–c all shared ~66% amino acid sequence identity with AtSOC1. MtSOC1a was ~66% identical to MtSOC1b and c, while the latter two proteins, encoded by adjacent genes, were 93% identical (Fig. 1A).

Phylogenetic analysis of the MIK domains of TM3-like proteins from temperate legumes (Medicago, pea, chickpea, and red clover), Arabidopsis, and soybean (Fig. 1B) and one with additional TM3 proteins (Dorca-Fornell *et al.*, 2011) (Supplementary Fig. S1A) indicated that all four Medicago TM3 proteins were in branch 1, suggesting that branch 2-type proteins had been lost or had diverged in Medicago. Branch 1 was subdivided into a SOC1-like clade with the three MtSOC1 proteins grouping with AtSOC1 and Medtr4g102530 grouping with other AGL14/19-like proteins (Fig. 1B). Within the SOC1 clade, the temperate legumes each had at least one SOC1a protein and one SOC1b/c protein, as recently observed using an IK domain phylogeny (Fudge *et al.*, 2018). These legume *SOC1a* and *SOC1b/c* type paralogues probably originated during the whole-genome duplication event that pre-dated speciation of legumes ~58 million years ago (Bell *et al.*, 2010; Young *et al.*, 2011; Fudge *et al.*, 2018). A more recent gene duplication event during speciation of the temperate legumes probably generated the two adjacent paralogues of the ‘*b/c*’ type gene that are only found in Medicago and alfalfa (Fudge *et al.*, 2018).

Based on our *in silico* analysis of normalized RNA Seq data from different tissues (MedicMine *Medicago truncatula* genome database) (Supplementary Fig. S1B), the Medicago TM3 genes have gene-specific expression patterns, but all share the feature of being predominantly expressed in vegetative tissues, as previously observed for the *MtSOC1* genes (Fudge *et al.*, 2018). *MtSOC1a* and *MtSOC1c* are expressed at similar levels in the aerial tissues; leaf blades and vegetative buds, while *MtSOC1b* has a different pattern of expression because it is not detected in leaf blades, and only very weakly in vegetative buds.

### RT–qPCR analysis of *MtSOC1* expression in Medicago

Recently, the expression of the *MtSOC1* genes was shown to be elevated by the environmental cues that induce flowering and regulated by *FTa1* (Fudge *et al.*, 2018). Our analysis by RT–qPCR of leaves and shoot apices in time courses, as opposed to a combination of total aerial tissues and apices in Fudge *et al.* (2018), gave similar results overall (Supplementary Figs S2–S4).

In different photoperiods, transcript of all three genes was more abundant in LDs than in SDs, with the greatest differential expression observed for *MtSOC1a*, which was highly elevated in the shoot apex of LD plants (Supplementary Fig. S2A, B). In the LD time course (Supplementary Fig. S2C, D), *MtSOC1a* transcript showed an elevation in the leaf and a more dramatic increase in the shoot apex, at 57 d old, prior to flowering. After flowering, expression dropped in the apex, but continued to rise in the leaf. There was a striking decrease in transcript levels in flower buds and it was further reduced in open flowers, as expected. *MtSOC1b* had a very different pattern of expression; its transcript was present at low levels during vegetative development, but it showed an abrupt increase after flowering (Supplementary Fig. S2E, F). There was a strong decline in *MtSOC1b* expression in flower buds, with even lower expression in open flowers. *MtSOC1c* transcript was present early in young plants and gradually increased through development in both leaves and apices (Supplementary Fig. S2G, H). At 57 d, a pronounced increase in *MtSOC1c* expression was observed in the apex, similar to *MtSOC1a*. It was weakly expressed in buds and was greatly reduced in open flowers, a feature it shares with the other *MtSOC1* genes.

In a vernalized long day (VLD) time course carried out after vernalzing germinated seeds (Supplementary Fig. S3), the expression patterns of the three *MtSOC1* genes and *MtPIM*, an *AP1* orthologue (Benlloch *et al.*, 2006; Cheng *et al.*, 2018), were similar to those observed in LDs, except that more *MtPIM* expression was detected in the leaves of VLD- than in those of LD-grown plants (Jaudal *et al.*, 2014). None of the four genes was expressed during cold treatment of germinated seeds. In a time course of vernalized *fta1* mutant and wild-type seedlings in LDs (Supplementary Fig. S4), *MtPIM* expression in the *fta1* mutant was barely detected, indicating dependence on *FTa1* (Laurie *et al.*, 2011)*. MtPIM* transcript levels eventually increased at flowering in the shoot apices, indicating that other factors apart from *FTa1* can regulate *MtPIM* expression (Supplementary Fig. S4A, B) (Laurie *et al.*, 2011). *MtSOC1a* showed a much slower rate of increase in the *fta1* mutant compared with the wild type, indicating that *FTa1* is important for the rapid increase of *MtSOC1a* prior to flowering in the wild type (Supplementary Fig. S4C, D). There was almost no increase in the expression of *MtSOC1b* and *MtSOC1c* in the leaves and apices of *fta1* mutants, indicating strong dependence of *MtSOC1b* and *MtSOC1c* on functional *FTa1*, in parallel with previous findings (Supplementary Fig. S4E–H) (Fudge *et al.*, 2018). These results indicate that *FTa1* is required for the correct temporal pattern of transcript accumulation of the *MtSOC1* genes in the wild type. Consistent with this, in *35S:FTa1* transgenic plants, there was a very large increase in the expression levels of *MtPIM* and strong elevation of the *MtSOC1* genes in the shoot apices (Supplementary Fig. S4J). In leaves, *MtSOC1a* was elevated, while only a modest increase was observed for *MtPIM* and *MtSOC1b*, and not much change for *MtSOC1c* (Supplementary Fig. S4I).

In summary, all the *MtSOC1* genes were expressed in SDs, but at a higher level in LDs, and all were dependent to varying degrees on *FTa1*. *MtSOC1a* and *MtSOC1c* both showed steep elevation in transcript levels prior to flowering in the shoot apex, implicating them as candidate regulators of the transition to flowering in Medicago.

### Overexpression of *MtSOC1a* accelerates wild-type Arabidopsis flowering time

**Fig. 2.**
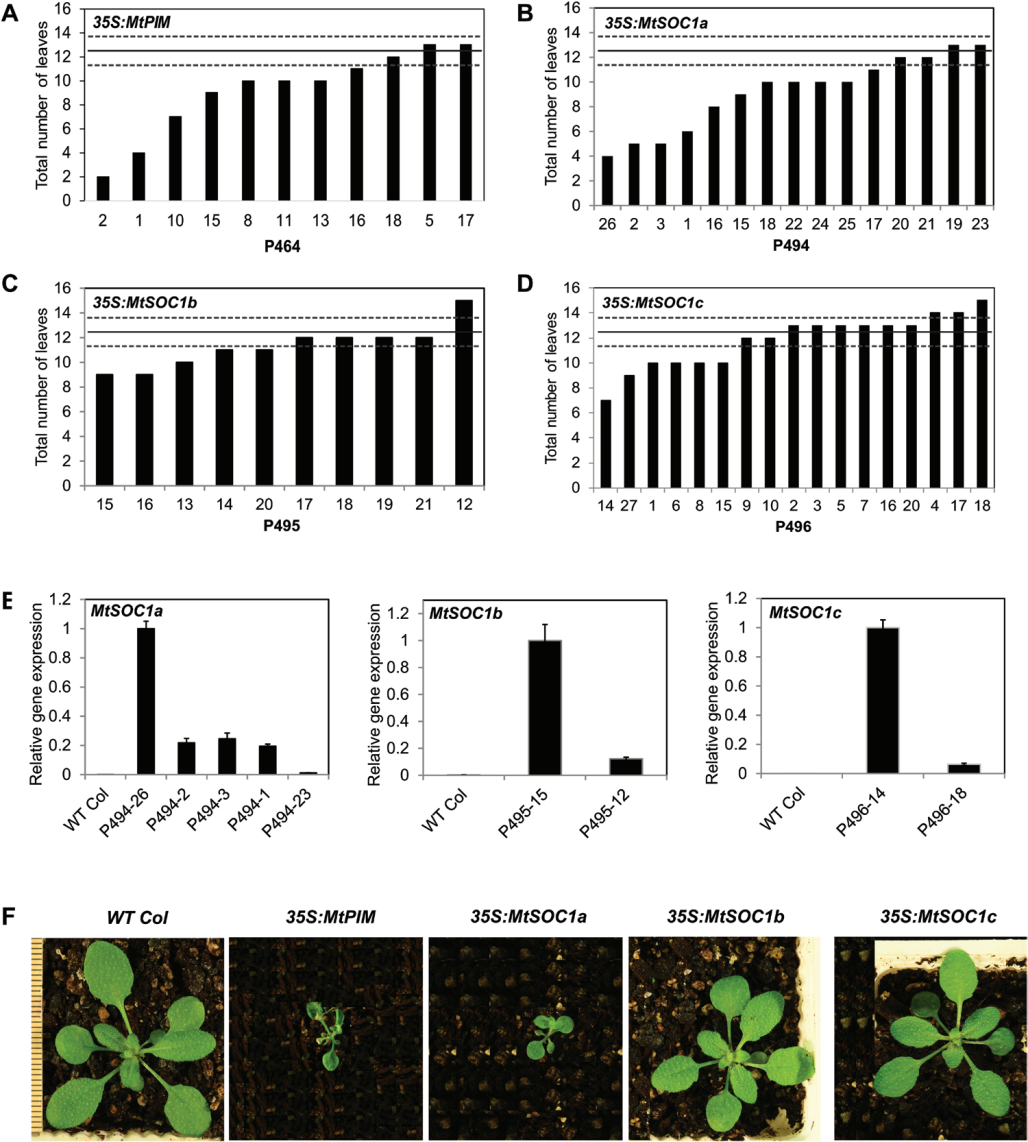
Flowering time and gene expression analyses of transgenic Arabidopsis plants with *35S:MtPIM* and *35S:MtSOC1* transgenes in long-day (LD) conditions. (A–D) Graphs showing the flowering time in LDs of transgenic T_1_ Col plants transformed with *35S:MtPIM* (A), *35S:MtSOC1a* (B), *35S:MtSOC1b* (C), and *35S:MtSOC1c* (D). Flowering time was measured as the total number of rosette and cauline leaves at flowering. The solid line represents the average flowering time of Arabidopsis wild-type Columbia, 12.5 ± 1.10 (t.SE) leaves (0.05) (dashed lines). (E) Gene expression of *MtSOC1a*, *MtSOC1b*, and *MtSOC1c* in representative T_1_ transgenic *35S:MtSOC1* Arabidopsis plants shown in (B–D) as compared with wild-type Col plants. Gene expression was determined in selected T_1_ plants with a range of flowering times using RT–qPCR and normalized to At2g32170. The data are shown as the mean ±SE of three PCR technical replicates. Data are presented relative to the plant with the highest transgene expression for each overexpression construct. (F) Photographs at the time of flowering of wild-type Col plants, a T_1_ plant from *35S:MtPIM* (P464-2), and representative T_2_ progeny from *35S:MtSOC1a* (P494-3), *35S:MtSOC1b* (P495-16), and *35S:MtSOC1c* (P496-27). The scale bar has lines 1 mm apart.

To investigate if *MtSOC1* or *MtPIM* genes can accelerate flowering in wild-type Arabidopsis, the Medicago genes were fused to the constitutive *Cauliflower mosaic virus* 35S promoter and transformed into Arabidopsis wild-type Col (Fig. 2).

The *35S:MtPIM* T_1_ transgenic plants had a range of flowering times, with the earliest flowering transformants flowering much more rapidly than the wild type (Fig. 2A). The early flowering *35S:MtPIM* plants were much smaller than the wild type, with tiny petioles, leaves, and flowers (Fig. 2F). Premature termination of the inflorescence was also observed due to formation of terminal flowers.

An extremely early flowering phenotype was also observed in four of 15 *35S:MtSOC1a* transgenic plants (Fig. 2B). The earliest flowering 35S:*MtSOC1a* transgenic plants were tiny at the time of flowering at 4–5 leaves (Fig. 2F). However, unlike *MtPIM* and *MtFULb* (Jaudal *et al.*, 2015), overexpression of *MtSOC1a* did not result in the formation of terminal flowers. Analysis of *MtSOC1a* transgene expression by RT–qPCR indicated that the earliest flowering line (P494-26) had the highest levels of transgene expression while the latest flowering line (P494-23), which flowered similarly to the wild type, showed very low levels of transgene expression (Fig. 2B, E). Apart from early flowering, other phenotypes were also observed in some *35S:MtSOC1a* plants including smaller plant size, looser flower structure, smaller flowers with shorter petals and sepals, and smaller and thinner siliques with low numbers of seeds that were dark in colour compared with the wild type (Supplementary Fig. S5).

The *35S:MtSOC1b* lines flowered similarly to Col wild-type plants (Fig. 2C), although transgene expression was confirmed in selected lines (Fig. 2E). The *35S:MtSOC1b* lines also had a similar plant architecture to the wild type (Fig. 2F).

The *35S:MtSOC1c* transgenic plants also flowered at a similar time to the wild type (Fig. 2D), with one exception, P496-14 with earlier flowering and elevated *MtSOC1c* transcript levels relative to the other transgenic plant tested (Fig. 2D, E). As observed in some *35S:MtSOC1a* lines (Supplementary Fig. S5), the earliest flowering *35S:MtSOC1c* plant was much shorter in size and produced smaller flowers with curved petals and shorter sepals and smaller siliques with lower yield and dark seeds compared with the wild type.

Recently, Fudge *et al.* (2018) showed that all three *MtSOC1* genes were able to promote flowering of *Atsoc1* mutants, in some cases close to wild-type levels when overexpressed, but none flowered earlier than wild-type plants. Here, of the three *MtSOC1* genes, overexpression of *MtSOC1a* appeared to have the strongest effect by dramatically accelerating flowering of four out of 15 transgenic plants compared with zero out of 10 of the *35S:MtSOC1b* lines and one out of 17 of the *35S:MtSOC1c* lines. The differences that we obtained with *35S:MtSOC1a* compared with Fudge *et al.* (2018) may be due to the two different genetic backgrounds, the numbers of plants analysed, relative level of expression of the transgenes, or growth conditions. MtSOC1a shares 66% amino acid identity with MtSOC1b and MtSOC1c, while the latter two are 93% identical to each other, which raises the possibility that a coding region difference between *MtSOC1a* and *MtSOC1b/c* might contribute to the difference between the genes in this assay.

### *In situ* hybridization with *MtSOC1a*

**Fig. 3.**
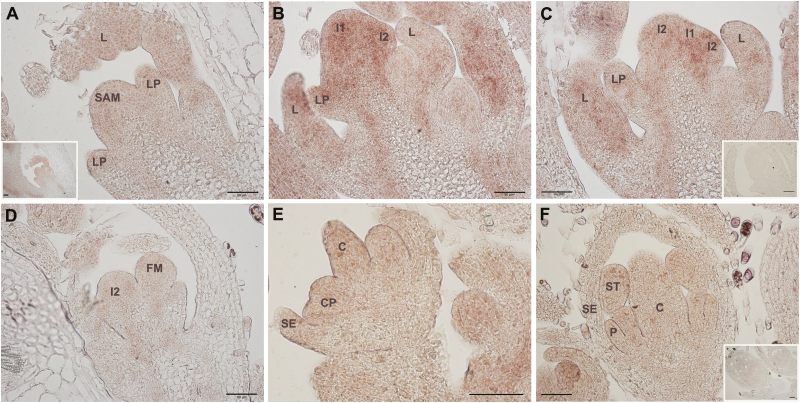
*In situ* localization of *MtSOC1a* transcripts through the floral transition in longitudinal sections of wild-type R108 plants grown in LDs, after 1 week of vernalization. (A) Vegetative shoot apical meristem (SAM) with leaf primordium (LP) and leaf (L). The inset shows the same section at a lower magnification. (B and C) SAM at the initial reproductive stage. Increased transcript abundance was detected in SAM, L, LP, and primary (I1) and secondary (I2) inflorescence meristems. (D–F) Weak *MtSOC1a* expression was detected during early stages of floral meristem development (D), during differentiation of floral organs (E), and in young flower buds (F). No expression was detected using a sense control *MtSOC1a* probe (inset images in C and F). C, carpel; CP, common primordium for petal and stamen; P, petal; SE, sepal; ST, stamen. The black scale bar represents 50 µm.

To analyse the function of *MtSOC1a* further, we examined the spatial expression pattern of *MtSOC1a* by *in situ* hybridization of longitudinal sections of shoot apices from 4-week-old VLD plants that had been vernalized for 1 week. These plants flowered when they were between 4 and 6 weeks old. In Arabidopsis, *SOC1* expression is detected in the shoot apical meristem (SAM) and leaf primordia during the transition to flowering, but not in young floral meristems, yet reappears in the centre of the floral meristem at a later stage of floral development (Borner *et al.*, 2000). *MtSOC1a* was detectable in the vegetative SAM and leaves (Fig. 3A). However, the *MtSOC1a* transcript level increased in the SAM, leaves, and primary (I1) and secondary (I2) inflorescence meristems at the initial reproductive stages (Fig. 3B, C), while weak expression was detected during the early stages of floral meristem development (Fig. 3D), during differentiation of floral organs (Fig. 3E), and in young flower buds (Fig. 3F).

In summary, our *in situ* hybridization result is analogous to our RT–qPCR data indicating that *MtSOC1a* transcript is expressed in Medicago during vegetative development and increases in level in the SAM during the transition to flowering.

### *Mtsoc1a* mutant plants are late flowering and have shorter primary stems

**Fig. 4.**
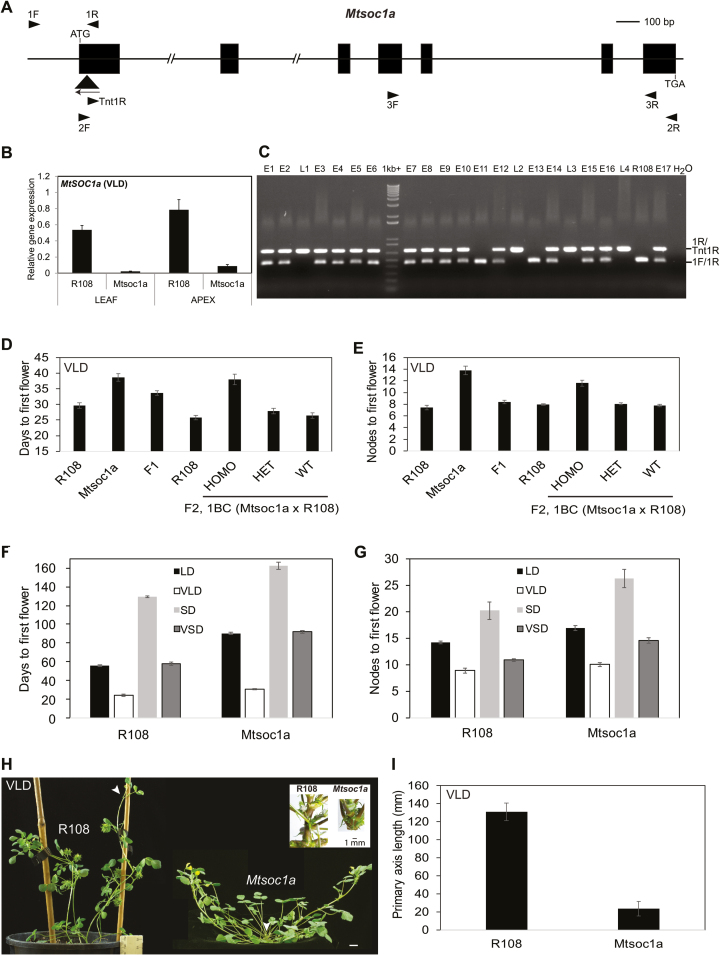
*Mtsoc1a* mutants have a recessive late flowering phenotype and reduced elongation of the primary stem. (A) Diagram of the *MtSOC1a* gene with the *Tnt1* insertion (black triangle) in the *Mtsoc1a* mutant. Exons are the black boxes and introns are thin lines. Arrowheads indicate primers. (B) Relative expression of *MtSOC1a* in 14-day-old R108 and *Mtsoc1a* seedlings in VLDs. Gene expression was determined using RT–qPCR with 3F and 3R primers, and the data are the mean ±SE of three biological replicates, normalized to Medicago *PP2A*. Data are presented relative to the highest value. Tissues were harvested 2 h after dawn. (C) Photograph showing PCR genotyping fragments from segregating VLD F_2_ plants. Plants were scored as early (E) (like R108) or late (L) flowering relative to R108. Three genotyping primers were pooled in the PCR. (D and E) Graphs showing the flowering time in vernalized LDs (VLDs) scored as the number of days to flowering (D) or the number of nodes on the primary axis at flowering (E) of the F_1_ progeny (*n*=14) from a backcross of *Mtsoc1a* mutants to wild-type R108 plants compared with *Mtsoc1a* mutants (self-cross) (*n*=10) and R108 plants (*n*=13), and the segregating F_2_ progeny from this backcross (*n*=206: *Mtsoc1a Tnt1* homozygotes, *n*=50; heterozygotes, *n*=111; wild-type segregants, *n*=45) with R108 wild-type control plants (*n*=28). The data are shown as the mean ± (t.SE) (0.05). (F and G) Graphs showing the flowering time in different conditions including short days (SDs) of *Mtsoc1a* mutants and R108 scored in either days (F) or nodes to first flower (G). The *Mtsoc1a* plants in LDs and VLDs were F_3_ plants after a backcross to R108. The mean ± (t.SE) (0.05) is presented (*n*=6–50). (H) Photographs of R108 wild-type plants with seed barrels and a prostrate, late flowering *Mtsoc1a* F_2_ homozygote with a very short primary axis taken at 45–47 d under VLDs. Arrowheads indicate the primary axis. Scale bar=1 cm. The inset image shows close-up photographs of primary shoot axes of 56-day-old LD wild-type R108 and *Mtsoc1a* F_3_ plants. (I) Graph showing the length of the primary axis of *Mtsoc1a* F_2_ homozygous mutants (*n*=39) after a backcross to R108 as compared with wild-type R108 (*n*=10). The measurements were taken at 45–50 d in VLDs and the data are shown as the mean ± (t.SE) (0.05).

To characterize *MtSOC1a* genetically, we used a mutant line (NF1705) segregating plants homozygous for a *Tnt1* retrotransposon insertion in the first exon of *MtSOC1a* (Fig. 4A) and with greatly reduced expression of *MtSOC1a* compared with wild-type R108 plants (Fig. 4B).

We crossed the homozygous mutants with wild-type R108 plants and grew an F_2_ population in VLDs. Out of 206 F_2_ plants, approximately a quarter (50) were *Mtsoc1a Tnt1* homozygotes and late flowering, approximately a half (111) were heterozygotes and flowered like wild-type R108, and approximately a quarter (45) were wild-type segregants and flowered like wild-type R108 (Fig 4C–E). Thus, there was 100% co-segregation between the homozygous *Tnt1* insertion in *MtSOC1a* and the late flowering phenotype. These results indicated that the *Tnt1* insertion in *MtSOC1a* was closely linked to the late flowering locus (within 1 cM) and that the mutation caused a recessive, late flowering phenotype.

In addition to their later flowering, *Mtsoc1a* mutant plants had a more prostrate appearance (Fig. 4H), because their primary shoot axis was shorter than that of the wild type in LDs and VLDs (Fig. 4H, I). This was associated with reduced internode elongation in the mutant (Fig 4H). Similar results were observed for a segregating population in LD conditions (Supplementary Fig. S6).

To investigate further the flowering response of the *Mtsoc1a* mutant, it was grown under different photoperiodic conditions with or without vernalization. The mutant flowered later than wild-type R108 plants under all conditions, in both days to flowering and in number of nodes on the primary shoot axis at the time of flowering (Fig. 4F, G). Thus, in summary, knock down of *MtSOC1a* in Medicago resulted in a delayed flowering time phenotype in the LD and SD conditions tested.

To analyse the molecular basis of the *Mtsoc1a* late flowering phenotype, candidate target genes were analysed using RT–qPCR in the leaves and shoot apices in VLDs (Supplementary Fig. S7). Only weak effects on gene expression were observed overall. *MtSOC1b* expression was slightly reduced in the leaves, while *MtSOC1c* had decreased expression in the leaves and shoot apices of *Mtsoc1a*. There was also a slight reduction in the transcript levels of *MtFULa-b* and *MtPIM* in the leaves of the mutants. All the other genes analysed including the five *FT-like* genes, *FTa1*, *FTa2*, *FTc*, *FTb1*, and *FTb2*; the candidate floral meristem identity gene, *LFY*; floral identity genes homologous to *SEPALLATA* (*SEP3a* and *SEP3b*); and candidate *AP2-like* genes, *TEMPRANILLO* (*TEM1*) and *TOE1-like* genes did not change much in either tissue of the *Mtsoc1a* mutants compared with the wild type.

Finally, we asked if mutations in the *MtSOC1b/c* genes can cause an altered flowering time or plant architecture phenotype. We could not identify a *Mtsoc1c* knock out mutant, but identified a *Mtsoc1b* line NF7041 with a homozygous *Tnt1* insertion in the second exon of *MtSOC1b* and gene expression knock down (Supplementary Fig. S8A, B). However, in contrast to *Mtsoc1a* mutants, the *Mtsoc1b Tnt1* insertion line did not display an altered flowering time phenotype or architecture in LD or VLD conditions (Supplementary Fig. S8C–F). A similar result was obtained with two other *MtSOC1b Tnt1* mutant lines (Fudge *et al.*, 2018).

### Overexpression of *MtSOC1a* in Medicago causes precocious elongation of the primary stem

**Fig. 5.**
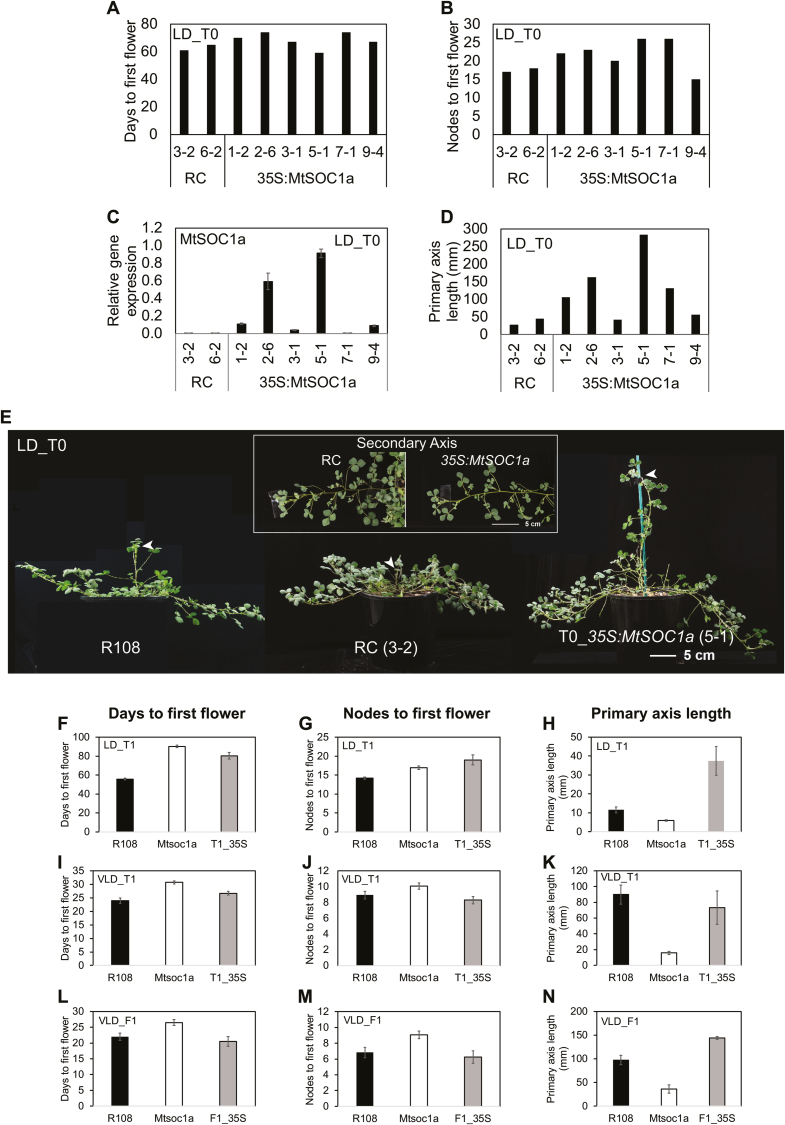
Overexpression of *MtSOC1a* in Medicago results in elongation of the primary stem. (A and B) Flowering time in either days (A) or nodes (B) to first flower of individual regeneration controls (RCs) and T_0_*35S:MtSOC1a* plants in LDs. (C) *MtSOC1a* transcript accumulation in leaves of RC and T_0_*35S:MtSOC1a* plants (32–39 d old) at ZT4. Gene expression was determined using RT–qPCR with data shown as the mean ±SE of three biological replicates, normalized to *PP2A* and relative to the highest value. (D) Graph showing the length of the primary axis of RC and T_0_*35S:MtSOC1a* plants (53–56 d in LDs). (E) Photographs of wild-type R108, RC, and T_0_*35S:MtSOC1a* plants in LDs, 55–57 d after transfer of germinated seeds (for R108) or regenerated plantlets to soil. Arrowheads indicate the primary axis. The inset image shows close-up photographs of secondary branches from the primary axis of RC and T_0_*35S:MtSOC1a* plants. (F–G, I–J) Graphs showing the flowering time of R108, *Mtsoc1a* mutants, and T_1_*35S:MtSOC1a* (T1_35S) in LDs and VLDs scored in either days (F, I) or nodes (G, J) to first flower. The mean ± (t.SE) (0.05) is presented (*n*=7–18). (H, K) Graphs showing the length of the primary axis of T_1_*35S:MtSOC1a* transformants in LDs (H) and VLDs (K) compared with *Mtsoc1a* and R108 plants. The measurements were taken at 33 d in LDs and 28 d in VLDs. The mean ± (t.SE) (0.05) is presented (*n*=7–18). The T_1_*35S:MtSOC1a* plants in (F–K) were progeny of T_0_*35S:MtSOC1a* (*5-1*). (L–N) Graphs showing the flowering time either in days (L) or nodes (M) to first flower and length of the primary axis measured at 28 d (N) in VLDs of the F_1_ progeny from a cross of T_1_*35S:MtSOC1a* to wild-type R108 (F1_35S) compared with *Mtsoc1a* and R108 plants. The mean ± (t.SE) (0.05) is presented (*n*=4–16). The *Mtsoc1a* plants in (F–N) were F_3_ homozygous mutants after a backcross to R108.

In order to investigate the effect of overexpressing *MtSOC1a* in Medicago, we transformed *Mtsoc1a* homozygous mutant leaf discs with the *35S:MtSOC1a* construct. Transformant plants were regenerated via somatic embryogenesis and planted into soil in LDs. The six independent *35S:MtSOC1a*/*Mtsoc1a* T_0_ lines had a range of gene expression levels, with some plants showing much higher expression of *MtSOC1a* than the regeneration controls (RCs) (Fig. 5C), but they all flowered at a similar time to *Mtsoc1a* RC at ~67 d with ~21 nodes on average (Fig 5A, B). Analysis of candidate flowering genes in the leaves and apices of transgenic plants also indicated that there was little change in their expression levels compared with RC plants (Supplementary Fig. S9).

Strikingly, however, four T_0_ transgenic plants had much longer primary shoot axes than RC and wild-type R108 plants (Fig. 5D, E). Thus, the short stem phenotype of the *Mtsoc1a* mutant was rescued by expression of *35S:MtSOC1a* in these transgenic plants. Plants, such as *35S:MtSOC1a* (*5-1*), with the highest level of *MtSOC1a* expression had the greatest primary shoot axis extension, indicating that primary stem elongation is sensitive to *MtSOC1a* levels. In contrast, the secondary branches of the *35S:MtSOC1a* transgenic plants only displayed a slight increase in internode elongation compared with RCs (inset, Fig. 5E).

Next, we analysed the T_1_ progeny of *35S:MtSOC1a* (*5-1*) in LD and VLD conditions. In LDs, the primary shoot was again much longer than in R108 wild-type controls, with little effect on flowering time (Fig. 5F–H). The stem elongation was striking because it was seen within the first month of growth and thus occurred well before the transition to flowering at ~80 d. These results indicate that it is possible to separate Medicago primary shoot elongation from the transition to flowering in LDs by ectopically overexpressing *MtSOC1a*.

In VLDs, in which R108 wild-type plants show much more rapid flowering and primary stem elongation than in LDs (Jaudal *et al.*, 2013), the *35S:MtSOC1a* (*5-1*) T_1_ plants had a similar primary shoot axis length to vernalized wild-type plants (Fig 5K). In terms of T_1_ flowering time, the delay in flowering seen in the *Mtsoc1a* mutant in VLDs was partially rescued (Fig. 5I, J). We observed that the *35S:MtSOC1a* T_1_ plants had longer primary stems in VLDs than in LDs, indicating that they were responsive to vernalization, but this was a reduced response compared with R108 (Fig. 5H, K).

To investigate the effect of the *35S:MtSOC1a* transgene in plants without the homozygous *Mtsoc1a* mutation, we crossed the *35S:MtSOC1a* (*5-1*) line with R108. The VLD F_1_ plants with the *35S:MtSOC1a* transgene flowered at a similar time to wild-type R108 plants, while their primary shoot lengths were slightly longer than those of vernalized wild-type plants (Fig. 5L–N). The same trend was observed in the LD F_2_ plants, in which the *35S:MtSOC1a* plants without the *Mtsoc1a* mutation had very long primary shoot axes but with flowering time similar to R108 plants. R108 flowered at an average of 56.4 ± 0.68 (t.SE) d (0.05) and the *35S:MtSOC1a* F_2_ segregants flowered at 55.7 ± 3.2 (t.SE) d (0.05).

In summary, the *35S:MtSOC1a* transgene partially complements the late flowering of the *Mtsoc1a* mutant, but does not cause further acceleration of flowering in the *MtSOC1a* wild-type background. In contrast, the *35S:MtSOC1a* transgene fully complements the short stem phenotype of the mutant and gives strikingly elongated primary shoot axes compared with the wild type, particularly in LD conditions.

### Increased length of the primary shoot axis in *35S:MtSOC1a* plants correlates with increased cell elongation

**Fig. 6.**
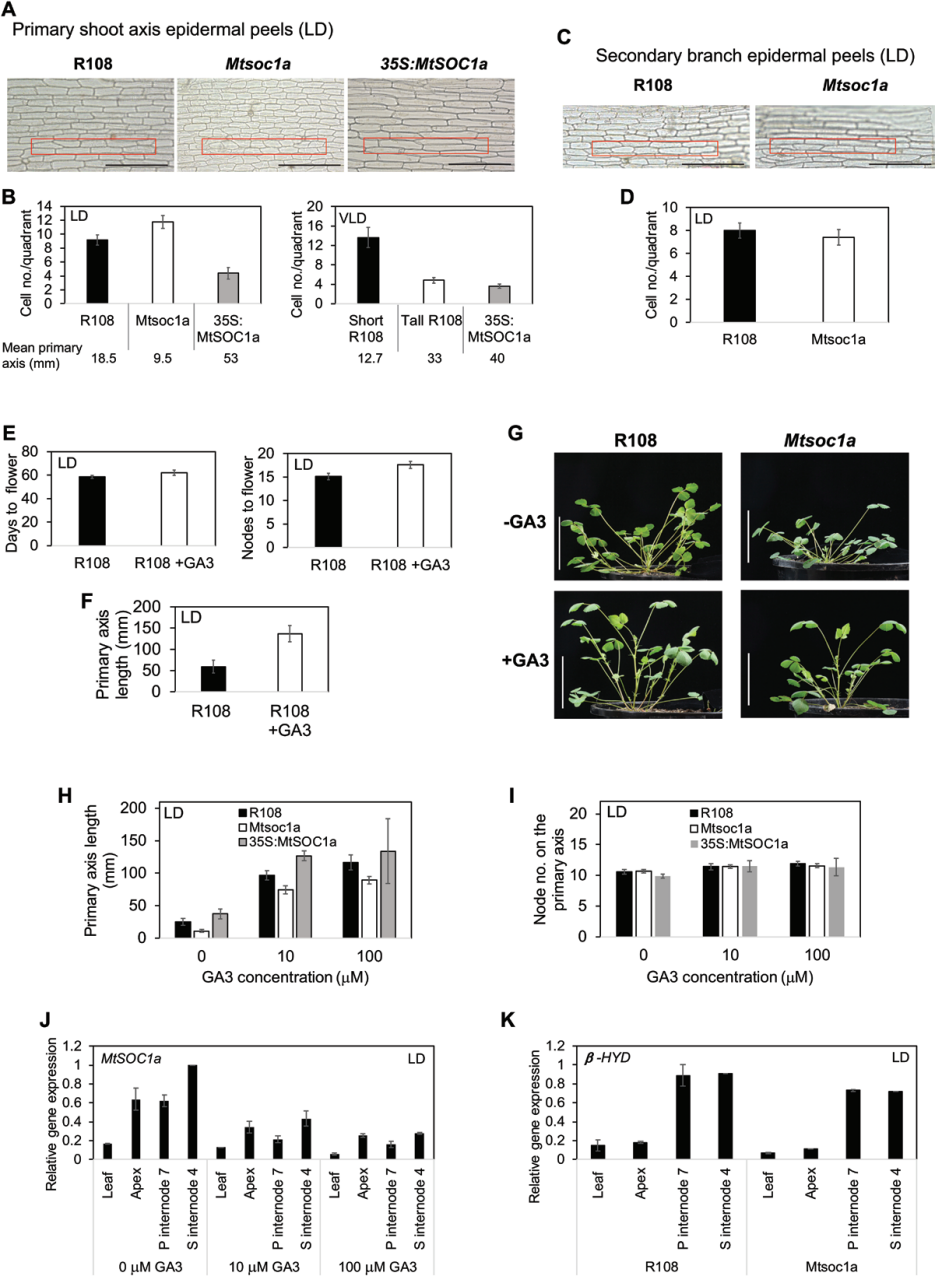
*MtSOC1a* promotes cell and internode elongation in the Medicago primary shoot axis, but is not required for the response to GA_3_ spray. (A) Photographs of epidermal peels taken from the primary shoot axis of R108 (internode 6), *Mtsoc1a* mutant, and a *35S:MtSOC1a* F_2_ plant (internode 6) (from a cross of T_1_*35S:MtSOC1a* to wild-type R108) of 31- to 32-day-old plants in LDs. (B) Graphs showing average cell number ± (t.SE) (0.05) within the boxed area (400 μm×50 μm) as shown in (A) on 9–12 epidermal peels of LD plants (left graph) and 5–8 peels of VLD plants (right graph). (C) Photographs of epidermal peels taken from internode 4 of secondary branches of 50-day-old LD R108 and *Mtsoc1a* plants. (D) Graph showing counts of the average cell number ± (t.SE)(0.05) on 5–6 epidermal peels within the boxed area. The scale bars in (A) and (C) are 200 µm. (E and F) Graphs showing the flowering time (E) scored in either days or nodes to first flower and the length of primary axis (F) at 67 d of LD R108 plants sprayed with 100 μM GA_3_ or control spray. The mean ± (t.SE) (0.05) is presented (*n*=8). (G) Photographs of 29-day-old LD plants sprayed with 100 μM GA_3_ (+GA3) or control spray (–GA) with a scale bar of 5 cm. (H and I) Graphs showing the lengths of primary shoot axes (H) and their node number (I) of 33-day-old LD R108, *Mtsoc1a*, and T_1_*35S:MtSOC1a* plants sprayed with 10 μM and 100 μM GA_3_ or control spray. Data are presented as the mean ± (t.SE) (0.05) (*n*=3–15). (J) Relative expression of *MtSOC1a* at ZT5 in the leaf, shoot apices of the primary and secondary branches (apex), internode 7 of the primary axis (P internode 7), and internode 4 of the longest secondary axis (S internode 4) of 42-day-old LD R108 plants sprayed with 10 μM and 100 μM GA_3_ or control spray. (K) Relative expression of *GIBBERELLIN 3 BETA-HYDROXYLASE 1-LIKE* (*β-HYD*, Medtr2g102570) in 42-day-old LD R108 and *Mtsoc1a* plants. Gene expression in (J–K) was determined using RT–qPCR, and data are the mean ±SE of two biological replicates, normalized to *PP2A* and presented relative to the highest value. The *Mtsoc1a* plants (C, D, G–K) were F_3_ homozygous mutants after a backcross to R108.

*35S:MtSOC1a* transgenic plants produced similar numbers of nodes to *Mtsoc1a* and wild-type plants in LDs (Fig. 5G), yet had much longer primary stems (Fig. 5H), suggesting that *35S:MtSOC1a* promotes internode elongation. To test if cell size was affected, we analysed cell counts within a set area (quadrant) on epidermal peels of stem internodes (Fig. 6A–D).

In LDs, the tall *35S:MtSOC1a* (*5-1*) plants had longer epidermal cells, thus fewer cells/quadrant, than the short *Mtsoc1a* mutants which had shorter cells (Fig. 6A, B). In VLDs, which promote primary stem elongation in the wild type (Jaudal *et al.*, 2013), the numbers of cells/quadrant were similar between a tall R108 plant and a *35S:MtSOC1a* (*5-1*) plant of similar height, while a younger, shorter R108 plant had smaller cells, thus more cells/quadrant (Fig. 6B). In contrast, the secondary branches of R108 and *Mtsoc1a*, which were of similar lengths, had similar epidermal cell sizes and thus similar cell counts/quadrant (Fig. 6C, D).

These results indicate that an increase in primary shoot axis length positively correlates with the increased epidermal cell size and that this is promoted by *MtSOC1a*.

### Wild-type and *Mtsoc1a* plants show increased shoot elongation in response to gibberellin

One of the classical roles of the hormone gibberellin (GA) is to promote stem elongation when applied to dwarf peas (Hedden and Sponsel, 2015). Thus, we hypothesized that the *Mtsoc1a* mutant with its shorter primary stem might have a defect in the GA response pathway. First, we tested the effect of applying 100 μM GA_3_ spray weekly to wild-type R108 plants in LDs. This led to a significant increase in primary shoot length (Fig. 6F, G). All plants had a similar node number at this stage (Fig. 6E), indicating that the GA_3_ spray had caused internode elongation, but did not promote flowering, with control plants flowering slightly earlier (Fig. 6E).

To compare the effect of GA_3_ application, we sprayed plants with GA_3_ (10 μM or 100 μM). The primary shoots were measured at 33 d old (Fig. 6H) when the plants had ~10 nodes on the primary shoot axis (Fig. 6I). Strikingly, all genotypes responded to GA_3_ with an overall increase in the primary shoot axis length that ranged from ~3-fold (*35S:MtSOC1a*), ~4-fold (R108), to ~10-fold (*Mtsoc1a*) with the 10 μM GA_3_ spray compared with the control spray, and with only a minor additional increase with the 100 μM spray. These results indicate that the *Mtsoc1a* short primary shoot phenotype does not appear to result from a defect in GA response in LDs.

The results also suggest that GA-mediated primary stem elongation in Medicago does not require *MtSOC1a*. Consistent with this, *MtSOC1a* expression in aerial tissues of wild-type plants, including stem internodes, was not elevated by GA_3_ application, rather it appeared to be slightly reduced, which may contribute to the slightly delayed flowering observed (Fig. 6J).

One alternative explanation for the short primary shoot stem of the *Mtsoc1a* mutant is that the mutant is defective in some aspect of GA production. Therefore, in an initial investigation, we examined the expression of a Medicago homologue of Mendel’s pea *Le* gene that is involved in pea internode elongation. The pea gene encodes a gibberellin 3 beta-hydroxylase involved in the production of bioactive GAs (Lester *et al.*, 1997). However, we did not see any differences in the transcript level of this gene between the *Mtsoc1a* mutant and wild type (Fig. 6K).

## Discussion

The transition to flowering in Medicago is promoted by vernalization and LD photoperiods, and is accompanied by changes in plant architecture including elongation of the primary stem (Chabaud *et al.*, 2006; Jaudal *et al.*, 2013). Our previous studies of Medicago *Tnt1* insertion mutants (Jaudal *et al.*, 2013, 2016; Yeoh *et al.*, 2013) identified *MtSOC1a* as a potential target of *FTa1*, a potent floral integrator gene (Laurie *et al.*, 2011). However, ours and others recent work indicate that *MtSOC1a* is one of three *SOC1-like* genes in Medicago that all depend on *MtFTa1* for the magnitude and timing of their expression and can partially complement the Arabidopsis *soc1* mutant, raising the possibility that they have redundant roles in flowering (Fudge *et al.*, 2018). We tried to identify *Tnt1* mutations in the three *MtSOC1* genes. We and others (Fudge *et al.*, 2018), identified several *Mtsoc1b* mutants, but none had a flowering time phenotype, while a *Mtsoc1c* mutant was not found. However, here we have identified a *Mtsoc1a* mutant with flowering time and growth phenotypes indicating that the *MtSOC1* genes do not have fully overlapping roles.

In Medicago, our spatial and temporal analysis of *MtSOC1a* gene expression in aerial tissues indicated that *MtSOC1a* transcript was highest in the SAM during the transition to flowering, suggesting a potential role in floral induction. Consistent with this, the timing and magnitude of *MtSOC1a* expression was also partially regulated by *FTa1* as *MtSOC1a* rapid accumulation was perturbed in the *fta1* null mutants. However, *MtSOC1a* transcript eventually reached relatively high levels in the leaves and apices of *fta1* mutants at flowering, indicating that other pathways or factors may turn on *MtSOC1a* expression in the absence of *FTa1*, as might be expected of a floral integrator gene (Fudge *et al.*, 2018). *MtSOC1a*, in turn, does not appear to be the only target of *FTa1* during floral promotion, because *fta1* mutants flower later than *Mtsoc1a* mutants and have lost their responsiveness to vernalization (Laurie *et al.*, 2011), unlike *Mtsoc1a* mutants.

The phenotype associated with a loss-of-function mutation in a *SOC1a-like* gene was not previously reported in legumes. Here we showed that *MtSOC1a* has the capacity to promote flowering in Medicago as shown in a *Tnt1* mutant line, which had knock down expression of *MtSOC1a* and was late flowering in all conditions tested. However, unlike in Arabidopsis, we did not observe precocious flowering in *35S:MtSOC1a* Medicago transgenic plants compared with wild-type plants.

*MtSOC1a* also has an important role in regulating primary shoot axis architecture. A striking feature of the *Mtsoc1a* mutants was their very short primary axis, which is a novel phenotype not observed in Arabidopsis *soc1* mutants. Overexpression of *MtSOC1a* reversed this phenotype. Indeed, it caused a dramatic increase in internode elongation and precocious elongation of the primary shoot axis in the transgenic plants, which was not associated with the transition to flowering and not observed in the wild type in LDs. These results indicate that *MtSOC1a* promotes internode elongation of the primary shoot axis of Medicago, consistent with its expression in stem internode tissue (Fig. 6J) (Fudge *et al.*, 2018).

Previously, we showed that VLD wild-type R108 plants had a much longer primary shoot axis compared with prostrate LD plants (Jaudal *et al.*, 2013). Our analysis of the architecture of the *spring* early flowering mutants (Jaudal *et al.*, 2013) also indicated that they had a much longer primary shoot axis compared with the wild type, while the late flowering *fta1* null mutants had a very short primary shoot axis (Laurie *et al.*, 2011) comparable with the *Mtsoc1a* mutants. These results suggest that the promotion of flowering by *FTa1* is normally tightly linked with primary shoot stem elongation. However, our analysis of the phenotype of *35S:MtSOC1a* transgenic plants provides the first evidence that primary shoot axis elongation and acceleration of flowering can be uncoupled in Medicago.

Further investigation of the *35S:MtSOC1a* plant phenotype indicates that the increase in internode elongation and thus primary axis height is correlated with an increase in epidermal cell length. This increase in cell length was also observed in wild-type R108 that have elongated primary axes in response to vernalization. Thus, elongation of the primary shoot axis in Medicago is probably due at least in part to increased cell expansion.

Because of the important role of GA in stem elongation in pea and because *AtSOC1* is downstream of GA-mediated floral promotion in Arabidopsis (Lee and Lee, 2010), we investigated if the short stem phenotype of *Mtsoc1a* mutants is due to a defect in responsiveness to GA. GA_3_ spray promoted stem internode elongation, but not early flowering in wild-type and *Mtsoc1a* plants in LDs. Thus, the short primary shoot phenotype of the mutant does not appear to result from a defect in GA response. This result also suggests that despite apparent similarities to the effect of *35S:MtSOC1a*, GA spray-mediated primary stem elongation in Medicago does not require functional *MtSOC1a*. Consistent with this, *MtSOC1a* expression was not elevated by GA_3_ application. Thus, *MtSOC1a* and GA may be involved in different pathways that regulate primary stem elongation in Medicago.

Ectopic expression of the other two *MtSOC1* genes, *MtSOC1b* and *MtSOC1c*, had little effect on flowering time of wild-type Arabidopsis plants in our study but, like *MtSOC1a*, they did partially complement the late flowering of the *Atsoc1* mutant, in some cases near to wild-type levels (Fudge *et al.*, 2018). However, *Mtsoc1b* mutants had no flowering or obvious architectural phenotypes, suggestive of functional redundancy (Fudge *et al.*, 2018). In addition, the Medicago *FUL-like* genes displayed quite similar patterns of expression and *FTa1* dependence (Jaudal *et al.*, 2015). Thus, gene editing approaches in Medicago (Meng *et al.*, 2017) targeting more than one of these genes at a time may serve to optimize future functional studies.

While many *SOC1-like* genes regulate flowering, others are known to affect other plant processes such as the duration of dormancy in kiwifruit (Voogd *et al.*, 2015). Overall, our study indicates that *MtSOC1a* regulates Medicago flowering time and primary shoot axis elongation, thus showing perhaps both conservation and divergence with Arabidopsis *SOC1* function. Since only weak changes in gene expression of candidate flowering time genes were observed in the *Mtsoc1a* mutant, genomic and biochemical analyses including hormone content may help to illuminate how *MtSOC1a* executes these roles.

## Supplementary Data

Supplementary data are available at *JXB* online.

Fig. S1. Phylogenetic tree of TM3 proteins and analysis of normalized Medicago RNA Seq data.

Fig. S2. Expression of *MtSOC1* genes in LDs and SDs, and overdevelopment in LDs.

Fig. S3. Expression of *MtPIM* and *MtSOC1* genes in VLDs.

Fig. S4. Expression of *MtPIM* and *MtSOC1* genes in *fta1*and *35S:FTa1* Medicago plants in LD vernalized seedlings.

Fig. S5. Plant architectures of transgenic Arabidopsis plants with *35S:MtSOC1* transgenes.

Fig. S6. Flowering time and stem lengths of a LD segregating population from R108×*Mtsoc1a.*

Fig. S7. Gene expression of candidate flowering genes in the *Mtsoc1a* mutant.

Fig. S8. *Mtsoc1b Tnt1* mutant flowered at the same time as the wild type.

Fig. S9. Expression of candidate flowering time genes in T_0_*35S:MtSOC1a* plants.

Table S1. List of primers.

## Supplementary Material

Supplementary Figures S1-S9 and Table S1
