# Supplementary material for: A SOC1-like gene MtSOC1a promotes flowering and primary stem elongation in Medicago
Source: J Exp Bot. 2018 Jul 26;69(20):4867–80. doi: 10.1093/jxb/ery284 (PMC6137972; doi:10.1093/jxb/ery284)
Supplement: Supplementary Figures S1-S9 and Table S1 [file ery284_suppl_supplementary_figures_s1-s9_table_s1.pdf]

Supplementary Data

Jaudal et al (2018) A *SOC1-like* gene *MtSOC1a* promotes flowering and primary stem elongation in Medicago

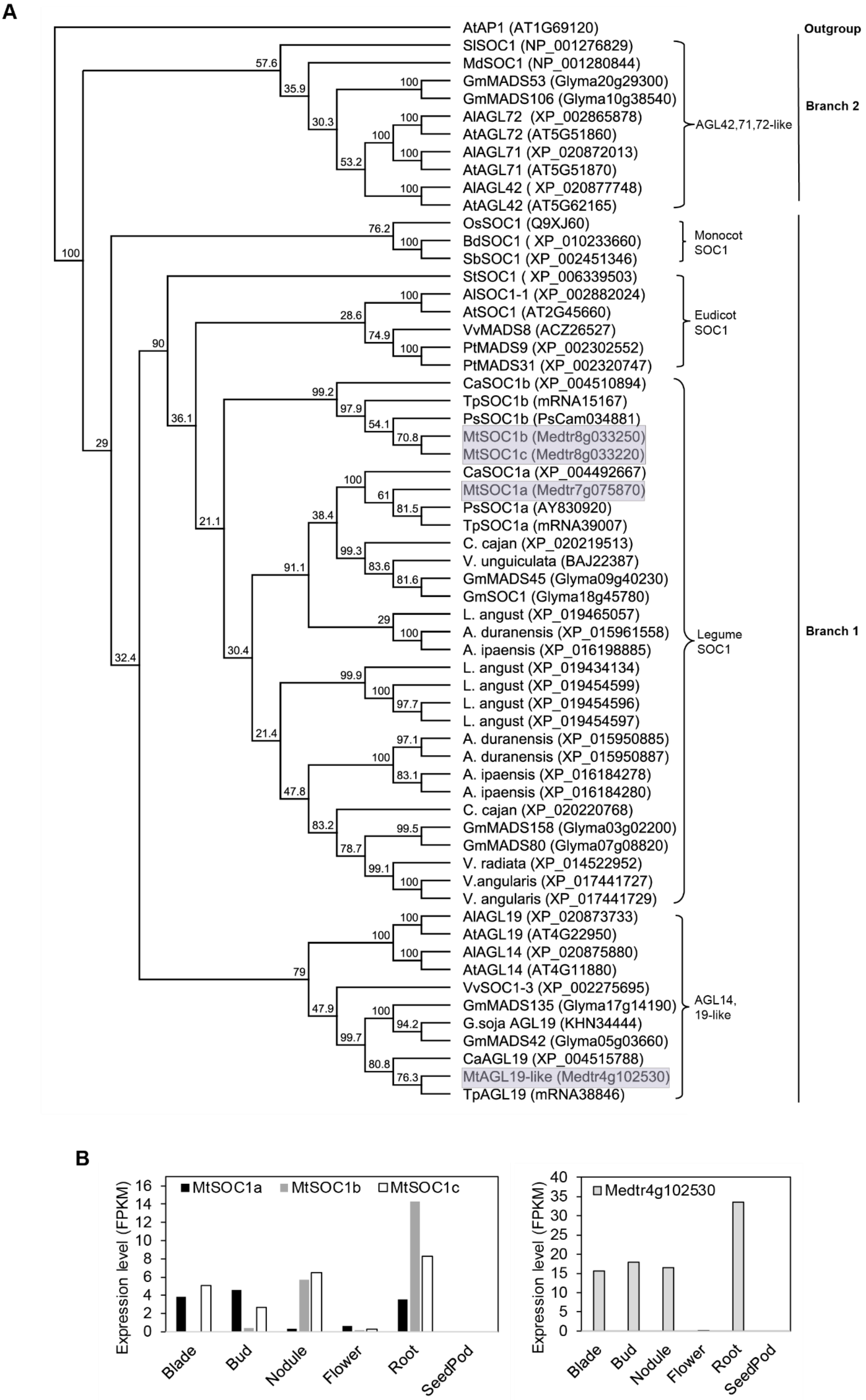
A
AtAP1 (AT1G69120)
SlSOC1 (NP_001276829)
MdSOC1 (NP_001280844)
GmMADS53 (Glyma20g29300)
GmMADS106 (Glyma10g38540)
AlAGL72 (XP_002865878)
AtAGL72 (AT5G51860)
AlAGL71 (XP_020872013)
AtAGL71 (AT5G51870)
AlAGL42 (XP_020877748)
AtAGL42 (AT5G62165)
OsSOC1 (Q9XJ60)
BdSOC1 (XP_010233660)
SbSOC1 (XP_002451346)
StSOC1 (XP_006339503)
AlSOC1-1 (XP_002882024)
AtSOC1 (AT2G45660)
VvMADS8 (ACZ26527)
PtMADS9 (XP_002302552)
PtMADS31 (XP_002320747)
CaSOC1b (XP_004510894)
TpSOC1b (mRNA15167)
PsSOC1b (PsCam034881)
MtSOC1b (Medtr8g033250)
MtSOC1c (Medtr8g033220)
CaSOC1a (XP_004492667)
MtSOC1a (Medtr7g075870)
PsSOC1a (AY830920)
TpSOC1a (mRNA39007)
C. cajan (XP_020219513)
V. unguiculata (BAJ22387)
GmMADS45 (Glyma09g40230)
GmSOC1 (Glyma18g45780)
L. angust (XP_019465057)
A. duranensis (XP_015961558)
A. ipaensis (XP_016198885)
L. angust (XP_019434134)
L. angust (XP_019454599)
L. angust (XP_019454596)
L. angust (XP_019454597)
A. duranensis (XP_015950885)
A. duranensis (XP_015950887)
A. ipaensis (XP_016184278)
A. ipaensis (XP_016184280)
C. cajan (XP_020220768)
GmMADS158 (Glyma03g02200)
GmMADS80 (Glyma07g08820)
V. radiata (XP_014522952)
V.angularis (XP_017441727)
V. angularis (XP_017441729)
AlAGL19 (XP_020873733)
AtAGL19 (AT4G22950)
AlAGL14 (XP_020875880)
AtAGL14 (AT4G11880)
VvSOC1-3 (XP_002275695)
GmMADS135 (Glyma17g14190)
G.soja AGL19 (KHN34444)
GmMADS42 (Glyma05g03660)
CaAGL19 (XP_004515788)
MtAGL19-like (Medtr4g102530)
TpAGL19 (mRNA38846)
Outgroup
Branch 2
AGL42,71,72-like
Monocot SOC1
Eudicot SOC1
Legume SOC1
Branch 1
AGL14, 19-like
B
MtSOC1a
MtSOC1b
MtSOC1c
Expression level (FPKM)
Blade
Bud
Nodule
Flower
Root
SeedPod
Medtr4g102530

Supplementary Fig. S1. Phylogenetic analysis of TM3-like sequences and *in silico* analysis of normalised RNA Seq data of *MtSOC1* and M*edtr4g102530* genes. **(A)** A consensus phylogenetic tree of TM3-like sequences from Arabidopsis, temperate and tropical legumes, and other plants based on MIK domains and rooted on Arabidopsis APETALA1 (AtAP1). The tree was generated using the Neighbor-Joining (NJ) method via bootstrap resampling with support threshold of 10%. The numbers indicate the bootstrap values based on 1000 replicates. Overall branch numbering is from Dorca-Fornell *et al.* (2011). The four Medicago proteins are highlighted in grey. Al: *Arabidopsis lyrata,* At: *Arabidopsis thaliana*, *Arachis ipaensis* and *Arachis duranensis* (peanut ancestors), Bd: *Brachypodium distachyon,* Ca: *Cicer arietinum* (chick pea), Cj: *Cajanus cajan* (perennial legume), Gm: *Glycine max* (soybean), *Glycine soja* (wild soybean), *Lupinus angustifolius* (lupin), Md: *Malus domestica* (apple), Mt: *Medicago truncatula*, Os: *Oryza sativa* (rice), Ps: *Pisum sativum* (pea), Pt: *Populus trichocarpa* (black cottonwood), Sb: *Sorghum bicolor*, Sl: *Solanum lycopersicum* (tomato), St: *Solanum tuberosum* (potato), Tp: *Trifolium pratense* (red clover), *Vigna angularis* (Adzuki bean), *Vigna radiata* (mungbean), *Vigna unguiculata* (cowpea), Vv: *Vitis vinifera* (common grape vine).

(B) Comparison of *MtSOC1* and *Medtr4g102530* gene expression in different tissues by *in silico* analysis of normalised RNA Seq data using the MedicMine *Medicago truncatula* genome sequence database.

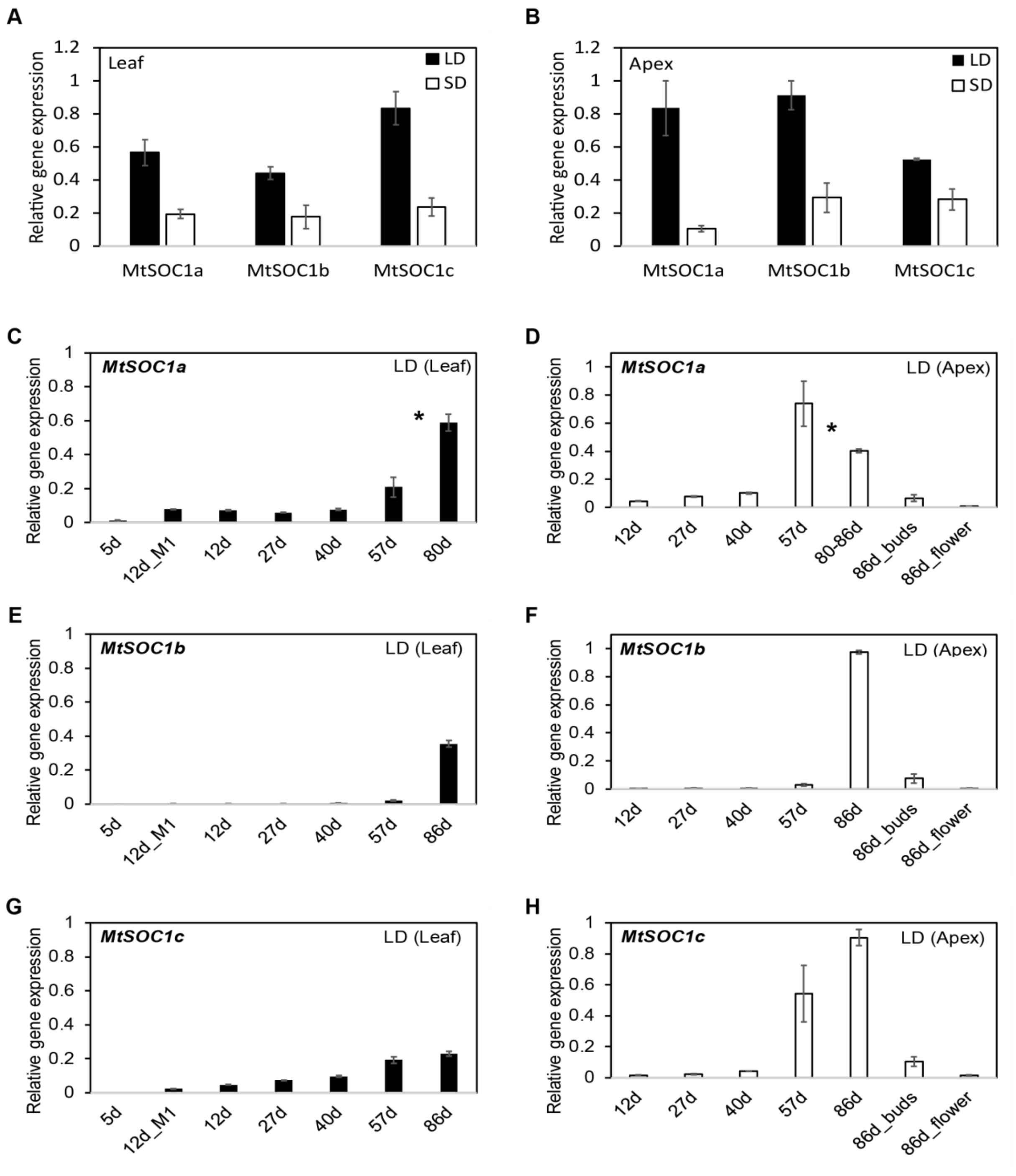

Supplementary Fig. S2. Expression of the three *MtSOC1* genes in Medicago wild type R108 plants under long day (LD) and short day (SD) photoperiods and during a developmental time course in LD. **(A-B)** Relative transcript abundance of *MtSOC1* genes in fully-expanded trifoliate leaves **(A)** and uppermost apical buds **(B)** of 14 day-old wild type R108 seedlings under LD and SD photoperiods. **(C-H)** Relative gene expression levels of the three *MtSOC1* genes in leaves (**C, E, G**) and uppermost apical buds, flower buds or open flowers (**D, F, H**) in days after planting in LD. The * in (**C-D**) indicate that the plants flowered with floral buds first visible at 69 days. Cotyledons were harvested at 5 days while monofoliate leaves (M1) or

trifoliate leaves were harvested at the remaining time points. Tissues in (**A-H**) were harvested two hours after dawn. Gene expression was determined using RT-qPCR and relative gene expression is shown as the mean ± se of three biological replicates, which were normalized to *PP2A*. The data is presented relative to the highest value for a specific gene.

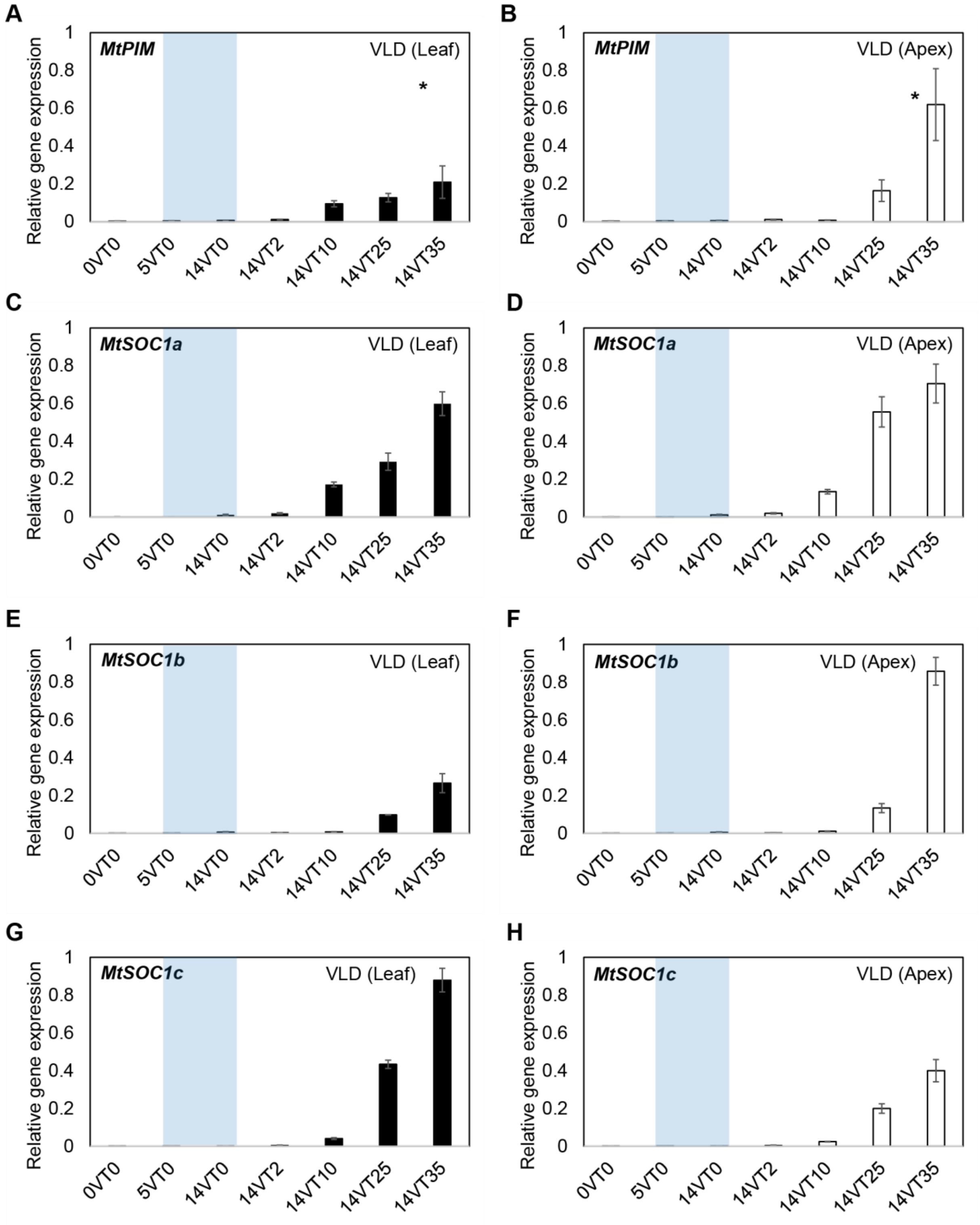

Supplementary Fig. S3. Expression of *MtPIM* and *MtSOC1* genes in a developmental time course in VLD by RT-qPCR. (**A-H)** Expression of *MtPIM* and *MtSOC1* genes in Medicago in a developmental time course in VLD. Relative gene expression levels of *MtPIM* and the three *MtSOC1* genes in whole seedlings, monofoliate leaves or trifoliate leaves (**A, C, E, G**) and

uppermost apical buds (**B, D, F, H**) before, during and after vernalisation are shown. After germination (0VT0), Medicago seeds were vernalised (shaded) by exposure to 14 days of cold (14V) at 4 °C in the dark, then planted in pots and transferred to warm (22 °C) LD conditions. The * indicates that plants had flowered with floral buds first visible at 32 days (T32) after planting of the vernalised seeds in the warm (14VT32). Tissues were harvested two hours after dawn. Whole seedlings were used for 0VT0 to 14VT0, whole aerial parts for 14VT2, and monofoliate leaves or trifoliate leaves and uppermost apical buds for 14VT10 and onwards. The grey shading indicates the 14-day vernalisation treatment. Gene expression was determined using RT-qPCR and the data are shown as the mean ± standard error (SE) of three biological replicates, which were normalized to *PP2A*. The data is presented relative to the highest value in all tissues for each gene.

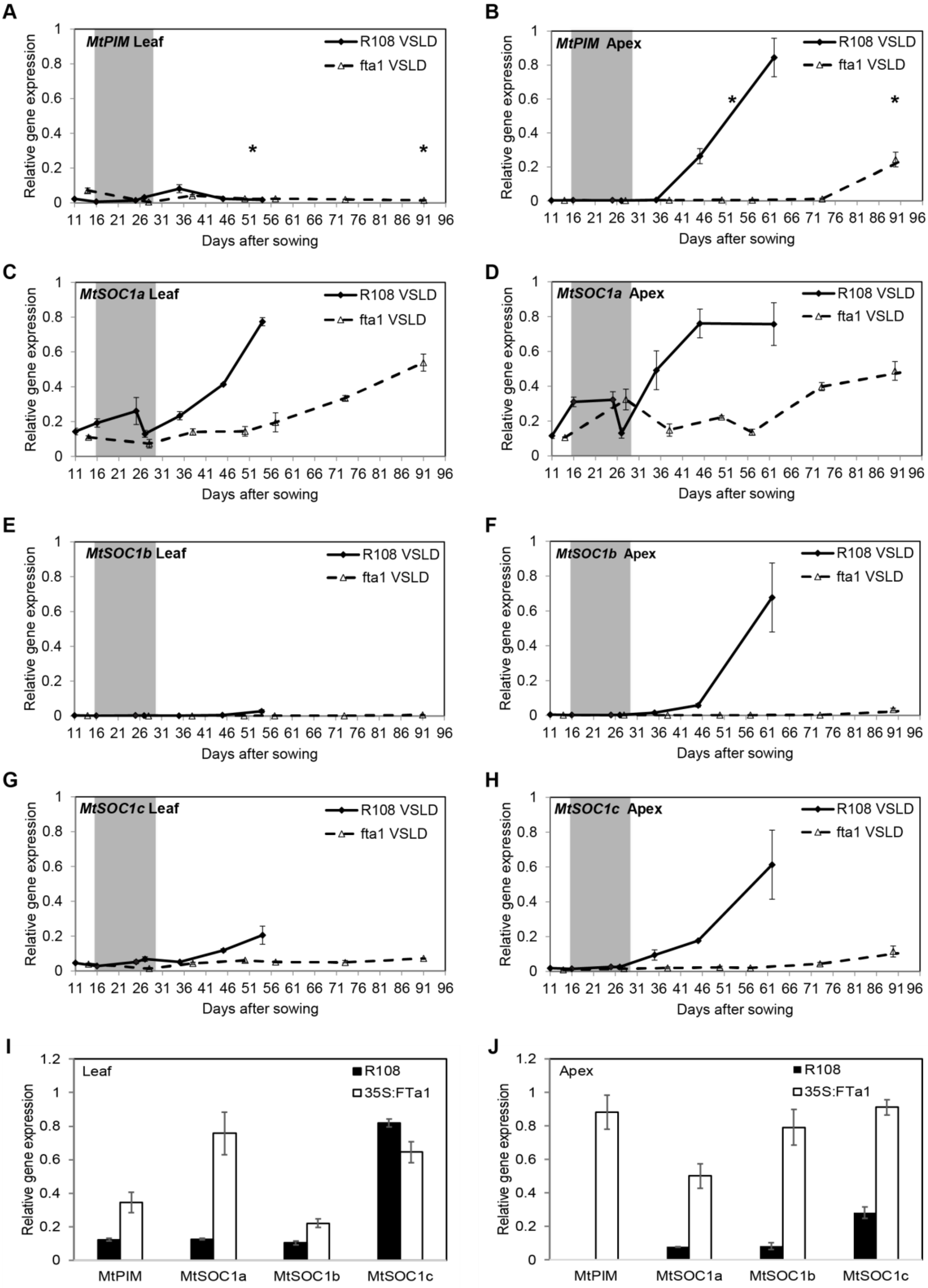

Supplementary Fig. S4. Expression of *MtPIM* and the three *MtSOC1* genes in *fta1*-null mutants and *35S:FTa1* transgenic Medicago R108 plants. (**A-H**) Comparison of expression of

MtPIM (**A-B**) and the three *MtSOC1* genes (**C-H**) in Medicago wild type R108 and mutant *fta1* plants in a developmental time course in LD after vernalisation of young seedlings (VSLD). Relative gene expression levels of *MtPIM* and the three *MtSOC1* genes in trifoliate leaves (**A, C, E, G**) and uppermost shoot apices (**B, D, F, H**) in seedlings during the 14 day vernalisation treatment (shaded) and after transfer to warm LD conditions are shown. The * shown in (**A-B**) indicate that plants had flowered with floral buds first visible on R108 plants at 52 days after planting and 91 days for the late-flowering *fta1* mutant. **(I-J)** Effect of ectopic expression of *FTa1* on *MtPIM* and the three *MtSOC1* transcript levels in *35S:FTa1* transgenic Medicago R108 plants in LD. Relative gene expression levels in trifoliate leaves (**I**) and uppermost apical buds (**J**) of *35S:FTa1* and wild type R108 14 day-old plants in LD are shown. Tissues were harvested at two hours after dawn (ZT2). Gene expression was determined using RT-qPCR and the data are shown as the mean ± se of three biological replicates, which were normalized to *PP2A*. The data is presented relative to the highest value over both tissues for each gene.

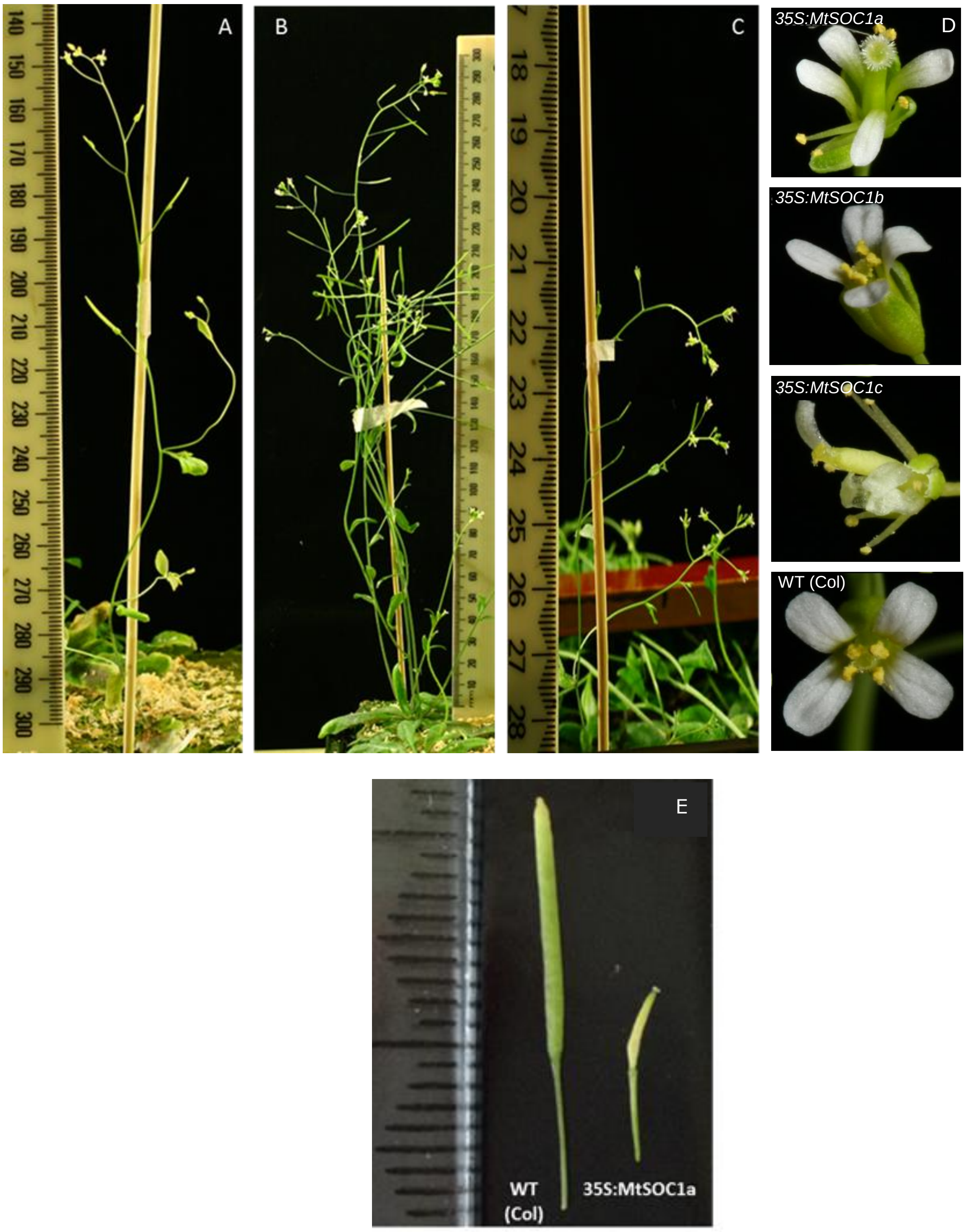

Supplementary Fig. S5. Plant, flower and silique architectures of transgenic Arabidopsis plants with *35S:MtSOC1* transgenes. **(A-C)** Photographs of *35S:MtSOC1a* (A), *35S:MtSOC1b* (B) and *35S:MtSOC1c* (C) transgenic plants. **(D)** Close-up photographs of flowers from *35S:MtSOC1a*, *35S:MtSOC1b* and *35S:MtSOC1c* transgenic plants and WT Col plants. **(E)** Photograph of siliques from WT Col and transgenic *35S:MtSOC1a* plants.

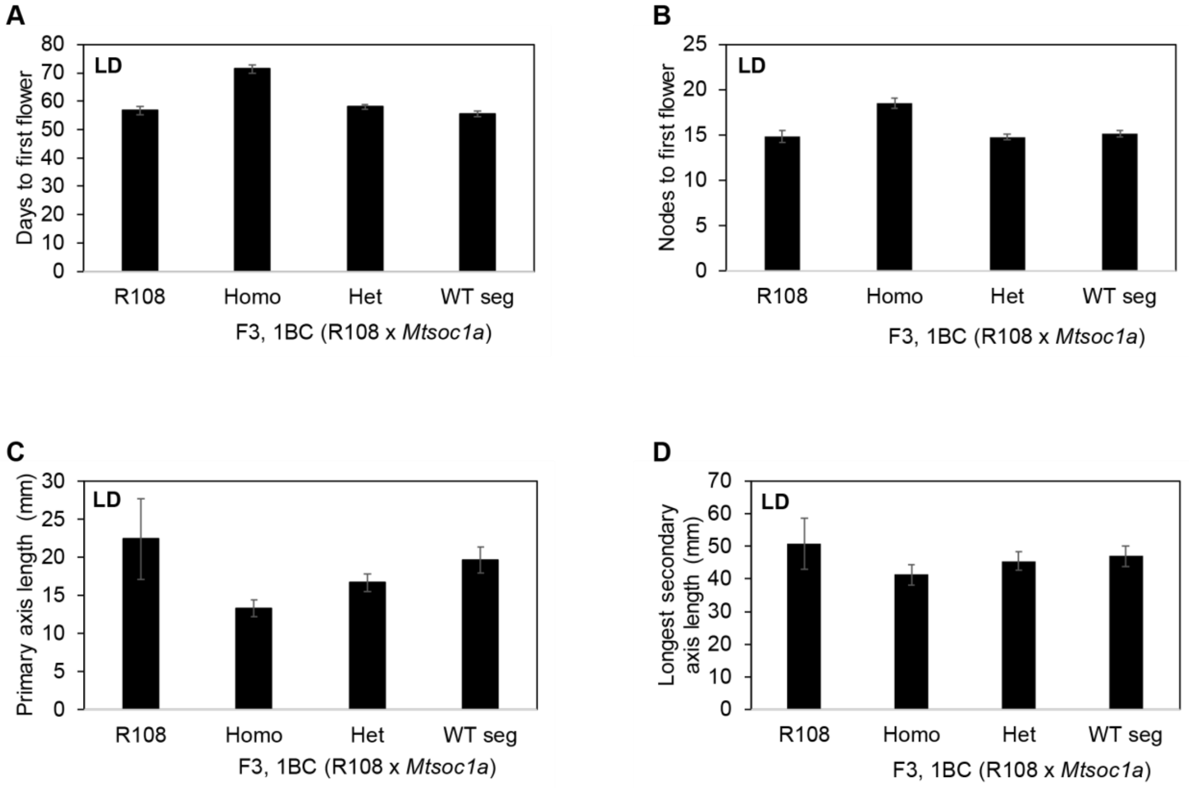

Supplementary Fig. S6. Flowering time and lengths of primary and secondary axes of a segregating population from a backcross of *Mtsoc1a* mutants to wild type R108 plants in LD. **(A-B)** Graphs showing the flowering time of R108 and F3 segregating population (R108 x *Mtsoc1a*) in LD scored in either days (A) or nodes to first flower (B). The mean ± t.se (0.05) is presented (n = 11-73). **(C-D)** Lengths of primary axis (C) and longest secondary axis (D) of plants shown in (A-B) measured at 31 day-old and presented as the mean ± t.se (0.05) (n=11-73).

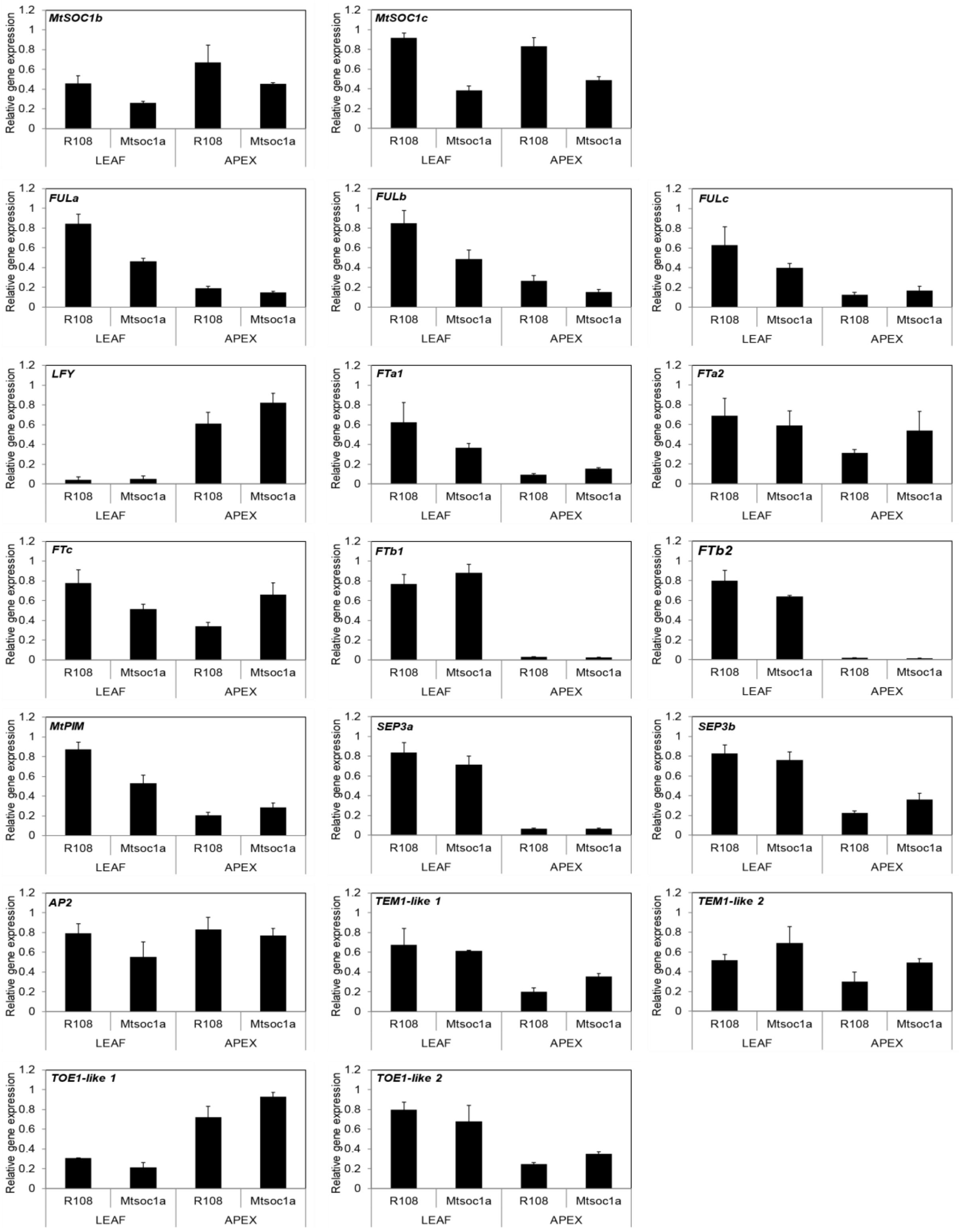

Supplementary Fig. S7. Gene expression of candidate flowering time and floral homeotic genes in the *Mtsoc1a* mutant. Relative transcript abundance was measured in the fully expanded trifoliate leaves and uppermost apical buds of 14 day-old wild type R108 and *Mtsoc1a* homozygous seedlings grown in vernalised long day (VLD) photoperiods. Relative gene expression was measured by RT-qPCR with normalization to *PP2A*. Data are the mean

± se of three biological replicates and relative to the highest value for each of the genes analysed. Tissues were harvested 2 h after dawn.

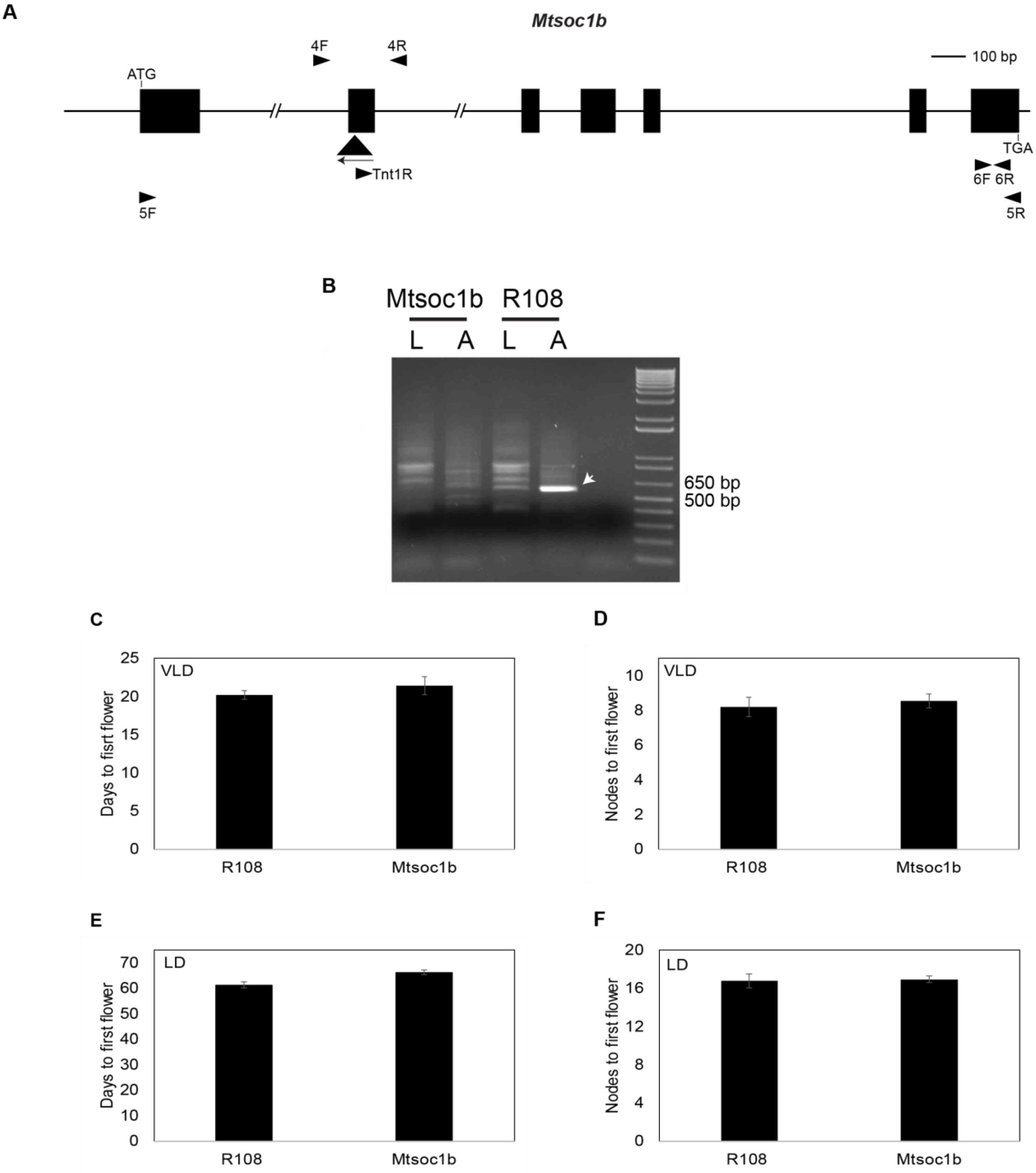

Supplementary Fig. S8. *Mtsoc1b Tnt1* mutants flowered at the same time as wild type R108. **(A)** Schematic diagram of the *MtSOC1b* gene and the position and orientation of *Tnt1* insertion in the *Mtsoc1b* mutant. Exons are shown as black boxes and introns as thin lines. A double line break indicates that the intron is larger than drawn. The arrowheads indicate the positions of primers. **(B)** Photograph of an agarose gel showing the presence of full-length cDNA (indicated by arrow) in wild type R108 but not in *Mtsoc1b* mutant plants. L: leaf; A: apex. (C-F) Graphs showing the flowering time of *Mtsoc1a* homozygous mutants as compared with wild type R108

scored in either days (C, E) or nodes to first flower (D, F) grown in VLD (C, D) and LD photoperiods (E, F). The mean ± t.se (0.05) is presented (n = 9-28).

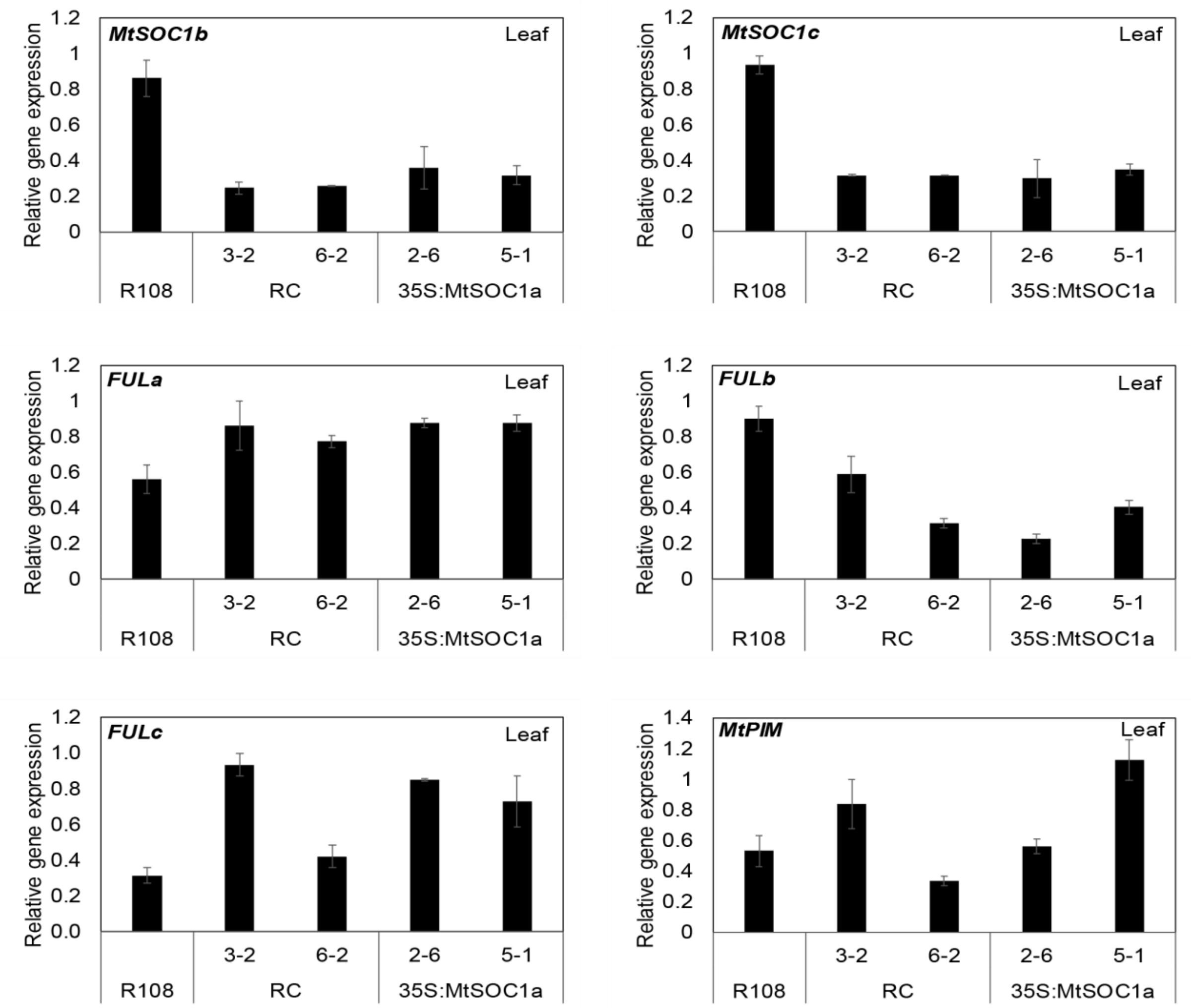

MtSOC1b
Leaf
MtSOC1c
FULa
FULb
FULc
MtPIM
Relative gene expression
3-2
6-2
2-6
5-1
R108
RC
35S:MtSOC1a

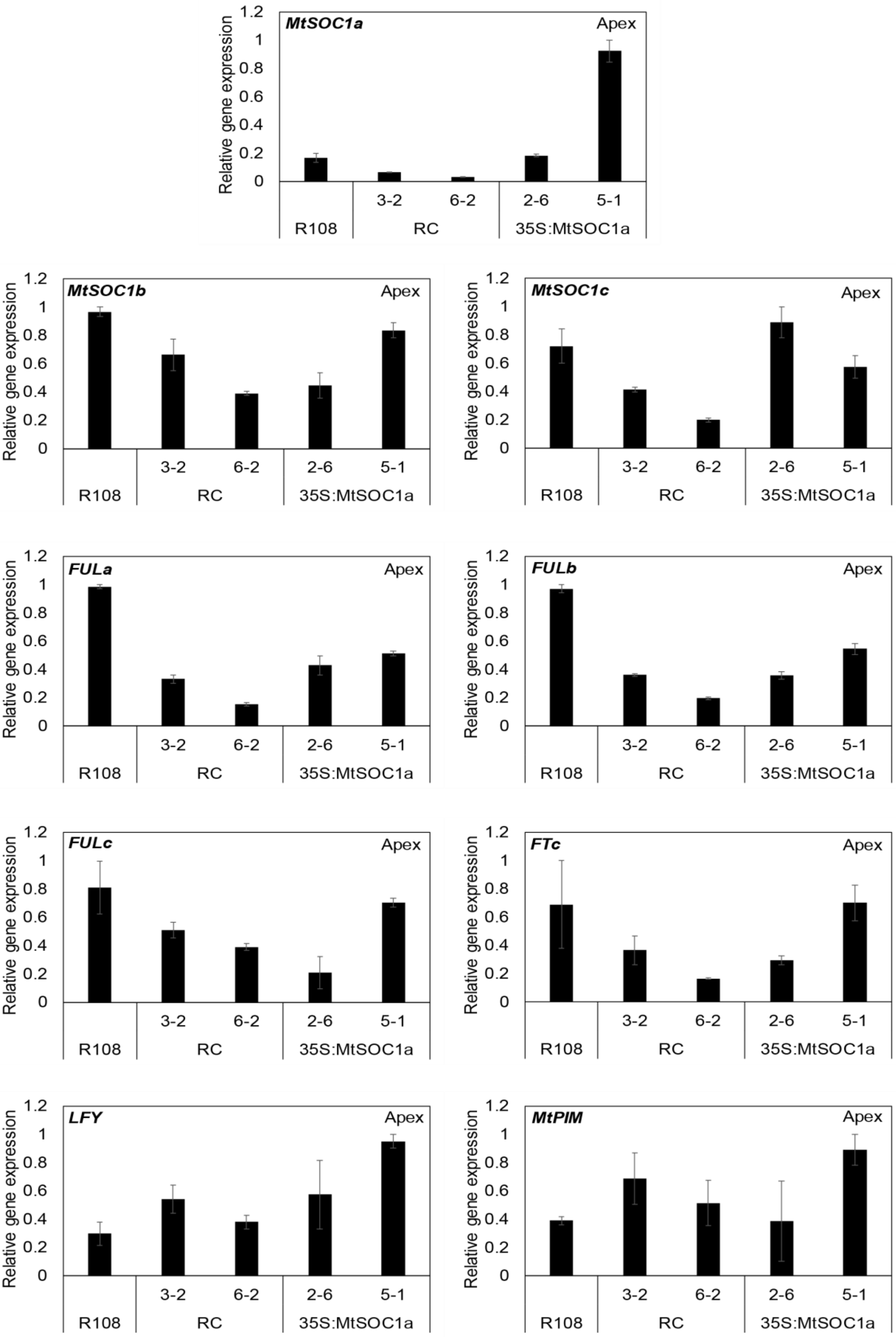
MtSOC1a
Apex
MtSOC1b
Apex
MtSOC1c
Apex
FULa
Apex
FULb
Apex
FULc
Apex
FTc
Apex
LFY
Apex
MtPIM
Apex
Relative gene expression
1.2
1
0.8
0.6
0.4
0.2
0
3-2
6-2
2-6
5-1
R108
RC
35S:MtSOC1a

Supplementary Fig. S9. Expression of candidate flowering time genes in leaves and apices of T0 *35S:MtSOC1a* transgenic plants. Gene expression was determined using RT-qPCR in the fully expanded trifoliate leaves (32-40 day-old) and shoot apices (77-86 day-old) of wild type R108, RC and T0 *35S:MtSOC1a* transgenic plants grown in LD. The data are shown as the mean ± SE of two-three biological replicates for R108 while two biological replicates for RC and T0 *35S:MtSOC1a* transgenic plants normalized to *PP2A* and presented relative to the highest value. The R108 tissues were harvested at ZT2 while the RC and T0 *35S:MtSOC1a* transgenic plants at ZT4.

Table S1. List of primers.

Gene	Purpose	Primer ID	Sequence
MtSOC1a	*Tnt1* line genotyping	1F	TGCTGCTGGTAGGTAAGAATCA
		1R	CTTCAACAAACCATTACGCC
	cloning cDNA and genotyping transgenic plants	2F	GGGGACAAGTTTGTACAAAAAAGCAGGCTT CGAAACAATGGTGAGAGGAAAGACAC
		2R	GGGGACCACTTTGTACAAGAAAGC TGGGTTCTTCAACTAGACCTGTGAAGTCC
	endogenous MtSOC1a expression in R108 or Total MtSOC1a expression (endogenous + transgene) in overexpression	3F	GCGTTGTTCGAGCAAGAAAGAATCAGGC
		3R	GGGGCTGCTTAGAGAGCCTGGCATTT
MtSOC1b	*Tnt1* line genotyping	4F	TGGAGCCTAAATCAGCCAGT
		4R	CGTCAAATTCTCCCCAAAAG
	cloning cDNA and genotyping transgenic plants	5F	GGGGACAAGTTTGTACAAAAAAGCAGGCTT GCTTCCTTTTGTTGCTGCA
		5R	GGGGACCACTTTGTACAAGAAAGCTGGGTT GAGACATTTGCCTCACCCT
	expression	6F	TCCAGAAACAAGATCAAGGCGCA
		6R	TGATGAGACATTTGCCTCACCCTT
MtSOC1c	cloning cDNA and genotyping transgenic plants	GW_MtSOC1c_F	GGGGACAAGTTTGTACAAAAAAGCAGGCT GAAGTTGCTTTTCTTGCTGCA
		GW_MtSOC1c_R	GGGGACCACTTTGTACAAGAAAGCTGGGT CCTCAACTAGCTACACCTTTGG
	expression	MtSOC1c-F	TGCCATGCTCGCAGAGAAGT
		MtSOC1c-R	TCAGTTTCCACATCTGAACTTGGACT
MtPIM	cloning cDNA and genotyping transgenic plants	GW_MtPIM_F	GGGGACAAGTTTGTACAAA AAAGCAGGCT ATGGGAAGGGGTAGGGTTCAGTT
		GW_MtPIM_R	GGGGACCACTTTGTACAAGAAAGCTGGGT TCAAAAGCATCCAAGATGGCAGG
	expression	MtPIM_qRT_F	GCACAATGGGAGCACCCAAACCA
		MtPIM_qRT_R	GCTTCCTCACGGTAATTGCCACCCA
At2g32170	expression	At2g32170-F	TCCTTTTTCATCGACACTGC
		At2g32170-R	CCATATGTGTCCGCAAAATG
FTa1	expression	RT_FTLa_F	GTAGCAGTAGGAATCCACTAGC
		RT_FTLa_R2	ACACTCACTCTCGGTTGATTTCC
FTa2	expression	FTa2-qRT-F	AAGTGGTAGCAGACCGAATC
		FTa2-qRT-R	CACCACCATTGGTAAC
FTc	expression	FTc-qRT-F	GTTATGGTGGACGCAGATGC
		FTc-qRT-R	CAAATCGATGAATCCCTGCT
FTb1	expression	FTb1-qRT-F	ATGAACCCTCTTGTGGTCTG
		FTb1-qRT-R	TGGATTGACTATTTGGGAAG
FTb2	expression	FTb2-qRT-F	ACAAATCCTCTTGTTGTTGG
		FTb2-qRT-R	TGAGTTGATTATTTGAGAGG
FULa	expression	MtFULa-TC182438-F	GGCCCAACTTGAGCAGCAAAATGAGG
		MtFULa-TC182438-R	TGGGCGTTGCCATGGGTTTGAC
FULb	expression	3_MtMADS5-qRT-F	AGAGCACGCAAAACTCAAGGCT
		3_MtMADS5-qRT-R	AGCTCTTTGAGACCTAAACCATCCAA
FULc	expression	MtFULc_new_F	AGGGCAAGGACATTGCAGGAGCA
		MtFULc_new_R	TGGTGGTAGCACCTCTGGCTGACAA
LFY	expression	MtLFY-Ex2-F	CACGTGGCAAAAAGAACGGT
		MtLFY-Ex2-R	TCACCGCGTTCCTTAGCAAT
AP2	expression	1_MtAP2-Ex9-F	ATTTTCCACATCTTCACCCCTC
		1_MtAP2-Ex10-R	TGCCATTTTTGTTGATCAGACATT
SEP3a	expression	MtSEP3a-F	GGAACACTTGCTATGTGAGGCAAACA
		MtSEP3a-R	TGGTTTTGACCTGGATGATGGCGT
SEP3b	expression	MtSEP3b-F	TGGGAAGAGGAAGAGTTGAGT
		MtSEP3b-R	AGAGCAACTTCAGCATCACAA
TEM1-like 1	expression	1_MtTEM1-Ex-F	TTGAGCCGGTTCAGATGGTT
		1_MtTEM1-Ex-R	TCCATTGCAGAACCCACCAA
TEM1-like 2	expression	2_MtTEM1-Ex-F	ATTGGTGTTGTCGGTGGTGA
		2_MtTEM1-Ex-R	ACCCCAAAAAGCCGAACCAT
TOE1-like 1	expression	1_MtTOE1-Ex9-F	TGGCATGTATCCCGCTTTCT
		1_MtTOE1-Ex10-R	AGTGACAAGGCCATGAGGTT
TOE1-like 2	expression	2_MtTOE1-Ex7-F	TGTCAATTTCAGACCGTCCCT
		2_MtTOE1-Ex8-R	CCAGCTTTTTCAAAGACGGGT
Tnt1	*Tnt1* line genotyping	Tnt1R	CAGTGAACGAGCAGAACCTGTG
β−HYD (Medtr2g102570)	expression	Medtr2g102570_Ex1-F	CGTTGGATCCCCTCTTGATCAT
		Medtr2g102570_Ex2-R	AGCCACATTAGTTTCCCTGCT
PP2A	expression	MtPP2A-F	GTGTTTTGCTTCCGCCGTT
		MtPP2A-R	CCAAATCTTGCTCCCTCATCTG
